# Axl receptor induces efferocytosis, dampens M1 macrophage responses and promotes heart pathology in *Trypanosoma cruzi* infection

**DOI:** 10.1038/s42003-022-04401-w

**Published:** 2022-12-29

**Authors:** Thaís S. Rigoni, Natália S. Vellozo, Kamila Guimarães-Pinto, Mariela Cabral-Piccin, Laryssa Fabiano-Coelho, Thayane C. Matos-Silva, Alessandra A. Filardy, Christina M. Takiya, Marcela F. Lopes

**Affiliations:** 1 Instituto de Biofísica Carlos Chagas Filho, Universidade Federal do Rio de Janeiro, Rio de Janeiro, RJ Brazil; 2 Instituto de Microbiologia Paulo de Góes, Universidade Federal do Rio de Janeiro, Rio de Janeiro, RJ Brazil

**Keywords:** Cell death and immune response, Apoptosis, Parasitic infection, Antimicrobial responses, Lymphocyte activation

## Abstract

Adaptive immunity controls *Trypanosoma cruzi* infection, but the protozoan parasite persists and causes Chagas disease. T cells undergo apoptosis, and the efferocytosis of apoptotic cells might suppress macrophages and exacerbate parasite infection. Nonetheless, the receptors involved in the efferocytosis of apoptotic lymphocytes during infection remain unknow. Macrophages phagocytose apoptotic cells by using the TAM (Tyro3, Axl, Mer) family of receptors. To address how the efferocytosis of apoptotic cells affects macrophage-mediated immunity, we employ here Axl receptor- and Mer receptor-deficient mouse strains. In bone marrow-derived macrophages (BMDMs), both Axl and Mer receptors play a role in the efferocytosis of proapoptotic T cells from *T. cruzi*-infected mice. Moreover, treatment with a TAM receptor inhibitor blocks efferocytosis and upregulates M1 hallmarks induced by immune T cells from infected mice. Remarkably, the use of Axl^−/−^ but not Mer^−/−^ macrophages increases T-cell-induced M1 responses, such as nitric oxide production and control of parasite infection. Furthermore, infected Axl^−/−^ mice show reduced peak parasitemia, defective efferocytosis, improved M1 responses, and ameliorated cardiac inflammation and fibrosis. Therefore, Axl induces efferocytosis, disrupts M1 responses, and promotes parasite infection and pathology in experimental Chagas disease. Axl stands as a potential host-direct target for switching macrophage phenotypes in infectious diseases.

## Abstract (web summary)

The tyrosine kinase receptor Axl induces efferocytosis, disrupts M1 responses, and promotes parasite infection and heart pathology in experimental Chagas disease.

## Introduction

American trypanosomiasis or Chagas disease, caused by infection with the protozoan *Trypanosoma cruzi*, affects over 6 million people worldwide^1^. Both *T. cruzi* infection and host immunity underlie the development of chronic disease, ranging from asymptomatic to cardiac or digestive forms of Chagas disease^2,3^. Despite morbidity and mortality associated with *T. cruzi* infection and advances in research, neither vaccines nor effective treatments for chronic Chagas disease are currently available^4,5^. *T. cruzi* parasites invade multiple cell types or are phagocytosed by macrophages, ultimately reaching the cytoplasm, where amastigote forms replicate. Then, parasites lyse host cells and spread through the blood to other tissues^3^. The intestines were identified as reservoirs and parasite sources for reinfection cycles in the heart^6^.

Both CD4 and CD8 T cells patrol host cells for parasite infection and play a protective role^2,7^. Cytotoxic CD8 T lymphocytes eliminate *T. cruzi-*infected target cells^8,9^, whereas the type-1 cytokine IFN-γ helps macrophages, which produce NO and mediate parasite killing^10^. In contrast, T helper 2 (Th2) cytokines and apoptotic cells downregulate macrophage activation and exacerbate parasite infection^11–13^. Overall, macrophage phenotypes can range from classically activated ‘M1’ macrophages to alternatively activated ‘M2’ macrophages, which express inducible nitric oxide synthase (iNOS) or arginase 1 (Arg1), respectively, as well as other hallmarks of their antimicrobial *versus* tissue repair roles^11,12,14–19^.

The death of T lymphocytes by apoptosis may shorten the breadth of immunity to infection in Chagas disease^20,21^. CD4 and CD8 T cells from *T. cruzi*-infected mice undergo apoptosis upon activation through the T-cell receptor (TCR):CD3 complex, Fas ligand (FasL)-Fas receptor interactions, and caspase-induced cell death^20,22–25^. We employed neutralizing anti-FasL^23^ and caspase inhibitors^24,25^ to unveil the molecular mechanisms underlying T-cell death in Chagas disease. Remarkably, treatment with either anti-FasL or the pan caspase inhibitor zVAD inhibited CD8 T-cell apoptosis during infection and enhanced T-cell and macrophage activation, allowing parasite clearance both in vitro and in vivo^23,24,26^. Recently, we addressed the outcomes of parasite infection in peritoneal macrophages cocultured with proapoptotic T cells generated during *T. cruzi* infection^26^. We found that Fas-mediated apoptosis of IFN-γ-producing CD8 T cells disabled macrophage-mediated immunity and promoted *T. cruzi* infection^26^. Conversely, inhibition of CD8 T-cell death skewed the macrophage phenotype towards an M1 effector response^26^.

In previous studies, we captured images of macrophages from *T. cruzi*-infected mice that phagocytosed apoptotic T cells^26,27^. Moreover, the phagocytosis of apoptotic cells (efferocytosis) by infected macrophages exacerbated *T. cruzi* infection in a TGF-β- and polyamine-dependent manner^13^. Therefore, inhibitors of apoptosis and efferocytosis are potential therapeutic tools to potentiate immune responses to some parasite infections^21,28–30^. Among the receptors involved in efferocytosis, TAM (Tyro3, Axl, and Mer) tyrosine kinase (TK) receptors^31,32^ are especially promising as molecular targets because new selective inhibitors for Axl or Mer signalling have been used in clinical trials to treat cancer^33^. Macrophages and dendritic cells (DCs) can phagocytose apoptotic cells through Mer and Axl receptors^34^ by binding to Protein S (ProS) and Gas6, which opsonize phosphatidylserine on the apoptotic cell membrane^31,32,35^. While Mer is important in constitutive efferocytosis, Axl expression is induced upon macrophage activation^36,37^. TAM-mediated efferocytosis of neutrophils has been previously shown not only to allow parasite transfer to DCs and macrophages in *Leishmania major* infection but also to negatively affect innate immunity mediated by macrophages and T-cell activation by DCs^38,39^.

By employing Axl- and Mer-deficient mouse strains, we investigated the role of individual TAM receptors on the phagocytosis of apoptotic T cells and T-cell control of M1/M2 phenotypes in *T. cruzi* infection. We uncovered a selective role of Axl in the suppression of T-cell-induced M1 macrophages in an experimental model of Chagas disease. Furthermore, reduced peak parasitemia and heart pathology indicate that Axl- but not Mer-deficient mice precociously controlled *T. cruzi* infection. We envision that Axl stands as a potential target to improve cellular immune responses to *T. cruzi* infection and that early control of parasite infection might prevent the development of chronic Chagas disease.

## Results

### Blockade of TAM receptors enhances the macrophage effector phenotype

**Fig. 1:**
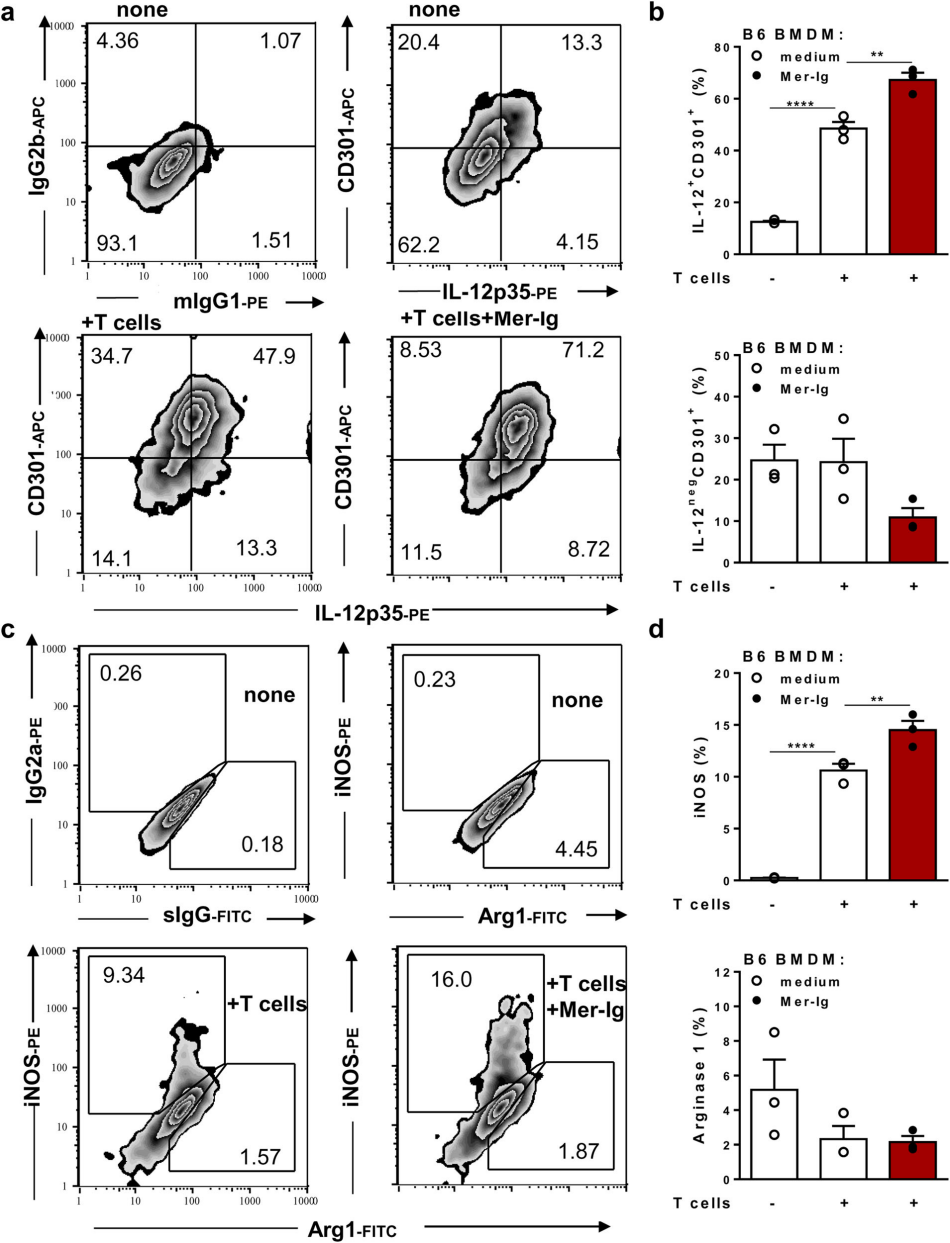
The blockade of TAM receptors improves M1 responses to immune T cells. BMDMs from B6 mice were treated with (closed circles) or without (open circles) the TAM inhibitor Mer-Ig and then cocultured in triplicate with T cells from *T. cruzi*-infected B6 mice (15 dpi) for 48 h. Macrophages were washed to remove T cells, collected, and stained. Gated F4/80^+^ cells were further analysed for **a**, **b** surface CD301 and intracellular IL-12p35 expression or control isotypes (IgG2b and mIgG1); **c**, **d** intracellular iNOS and Arg1 expression or isotype controls (IgG2a and sIgG). **b**, **d** The results are expressed as the means and SEM of *n* = 3 technical replicates and represent two independent experiments. Significant differences are indicated for ^**^P < 0.01, and ^****^P < 0.0001, as analysed by one-way ANOVA followed by Bonferroni posttest of selected pairs of data (macrophages cultured alone *versus* those cocultured with T cells; macrophages cocultured with T cells in the absence of treatment *versus* those treated with Mer-Ig).

To investigate how efferocytosis affects immunity induced by interacting T cells and macrophages, we cocultured bone marrow-derived macrophages (BMDMs) with splenic T cells from infected mice, thereby mimicking the immune milieu in *T. cruzi* infection (Supplementary Fig. 1a). Splenocytes obtained during acute infection provided both proapoptotic T cells (see below) and in vivo-primed IFN-γ-producing T cells for macrophage activation (Supplementary Fig. 1b). T cells from infected mice stimulated the production of the M1 chemokine CXCL9 in cocultured infected or noninfected BMDMs, and substantially increased NO production by infected macrophages (Supplementary Fig. 1b). We hypothesized that the inhibitory signalling induced through TAM receptors upon the uptake of apoptotic cells might counteract T-cell-induced M1 responses and hamper macrophage-mediated immunity. First, we employed the TAM receptor inhibitor Mer-Ig, which binds to the bridging molecules ProS and Gas6 on the apoptotic cell surface and prevents TAM-mediated efferocytosis (schematically represented in Supplementary Fig. 1c). Noninfected BMDMs from naïve B6 mice and T cells from infected mice were cocultured in the absence or presence of the TAM inhibitor Mer-Ig, as depicted in Supplementary Fig. 1d. After 48 h, the functional phenotypes of F4/80^+^ macrophages were analysed by using iNOS and IL-12p35 as M1 markers and arginase 1 (Arg1) and MGL (CD301) expression for M2 phenotyping (Fig. 1). In the absence of T cells, B6 macrophages showed a mixed phenotype: a fraction of cells expressed the M2 marker CD301 alone, whereas other cells coexpressed IL-12p35 and CD301 markers (Fig. 1a). Coculture with T cells from infected mice increased the percentages of CD301^+^IL-12^+^ cells (Fig. 1a, b) and induced iNOS expression (Fig. 1c, d). Moreover, treatment with both T cells and the TAM inhibitor further upregulated the percentages of CD301^+^IL-12^+^ cells, iNOS expression (Fig. 1a–d), and CXCL9 response (Supplementary Fig. 2b). In T-cell-macrophage cocultures from Th2-prone BALB/c mice, treatment with the TAM inhibitor also increased the expression of M1-phenotype hallmarks, such as interleukin (IL)-12 secretion, iNOS expression, and NO production (Supplementary Fig. 2). Therefore, the use of the TAM receptor inhibitor shifted macrophages expressing mixed phenotypes towards an M1-like effector phenotype. These results suggest that one or more TAM receptors might downmodulate the M1 phenotype induced by T cells from *T. cruzi*-infected mice.

### TAM receptor expression by B6 macrophages

**Fig. 2:**
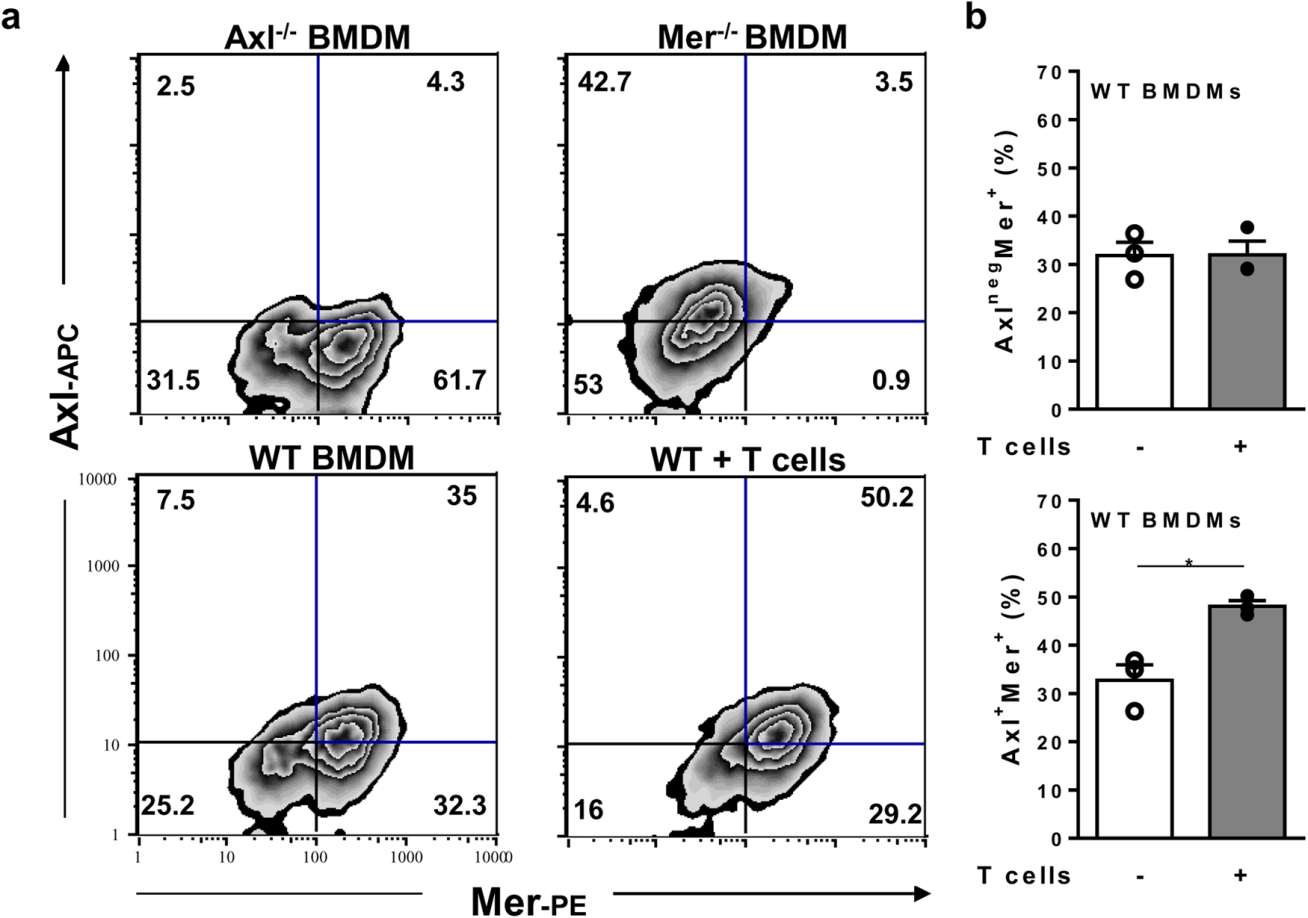
Macrophage expression of Axl and Mer receptors. **a**, **b** BMDMs from WT, Axl^−/−^ and Mer^−/−^ mice were cultured in triplicate in medium only (open circles) or with T cells from infected WT mice (closed circles) for 48 h and then analysed by flow cytometry. Macrophages were stained for F4/80, Axl, and Mer expression. **a** Analyses employed F4/80^+^ cells from Axl^−/−^ and Mer^−/−^ mice as negative controls for Axl and Mer expression. **b** The results are expressed as the means and SEM of *n* = 3 technical replicates and represent two independent experiments. Significant differences between macrophages cultured alone *versus* those cocultured with T cells are indicated for ^*^P < 0.05, as analysed by unpaired Student’s *t*-test.

To dissect the individual roles of the TAM receptors Axl and Mer on the immune responses to *T. cruzi* infection, we employed Axl- and Mer-deficient mice (Fig. 2a). Genetic ablation of *Axl* or *Mertk* was ascertained by polymerase chain reaction (PCR) (not shown). Then, we assessed Axl and Mer expression in F4/80^+^ BMDMs from WT (B6) mice under our culture conditions (Fig. 2a). Most unstimulated WT macrophages constitutively expressed the Mer receptor, whereas approximately 50% of Mer^+^ cells coexpressed the Axl receptor (Fig. 2a, b). Moreover, coculture with T cells from infected mice increased Axl^+^Mer^+^ cells to approximately 50% of all macrophages (Fig. 2a, b). However, more than 80% of macrophages expressed Mer at high levels, whereas only approximately 50% also expressed Axl in cocultures (Fig. 2a, b).

### TAM receptors induce T-cell efferocytosis by macrophages

Next, we analysed how the absence of Mer or Axl in macrophages would affect the efferocytosis mediated by BMDMs cocultured with T cells from *T. cruzi*-infected mice. Compared with normal mice, infected mice show increased frequencies of CD8 T cells^20,24^, which express a proapoptotic phenotype^40^ during acute infection (Supplementary Fig. 3a, b). In comparison with freshly isolated cells, CD8 T cells from infected mice showed increased staining with the apoptotic cell markers 7-aminoactinomycin D (7-AAD) and Annexin V (AnV) after 48 h-culture with macrophages (Supplementary Fig. 3b). For simplicity, we excluded 7-AAD and AnV-negative cells (alive cells) from the gate and considered all CD8^+^ cells dead within the gate. Stimulation with anti-CD3, used here only as a positive control for apoptosis induction^26^, yielded increased cell death in CD4 and CD8 T cells, most of which were late apoptotic (AnV^+^7-AAD^+^) cells (Supplementary Fig. 3b).

**Fig. 3:**
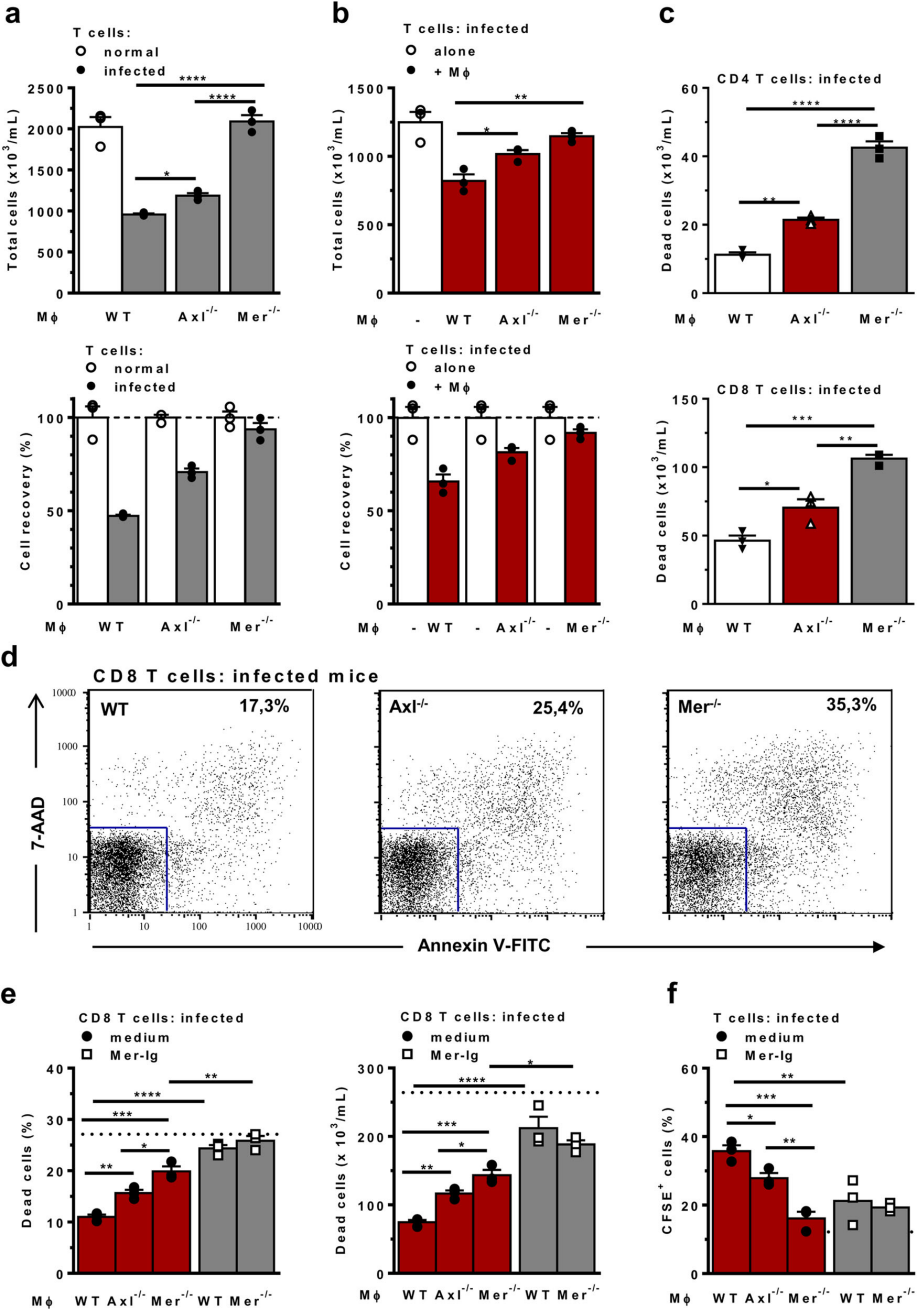
TAM receptor-mediated efferocytosis of proapoptotic T cells from infected mice. **a** BMDMs from WT, Axl^−/−^ and Mer^−/−^ mice were cocultured in triplicate with splenic T cells from normal (open circles) or *T. cruzi*-infected (closed circles) WT mice. After 48 h, nonadherent cells were counted, and cell recovery was calculated by using naïve T cells as controls. **b** T cells from infected mice were cultured in the absence (open circles) or presence (closed circles) of BMDMs from WT, Axl^−/−^ and Mer^−/−^ mice. Cell recovery was calculated based on T cells cultured in the absence of macrophages as 100% controls. **c** T cells from infected mice were cultured in the presence of BMDMs from WT (closed triangles), Axl^−/−^ (open triangles) and Mer^−/−^ (closed squares) mice. **c**, **d** T lymphocytes were labelled with anti-CD4, anti-CD8, Annexin V, and 7-AAD for evaluation of AnV^+^ and 7-AAD^+^ dead cells. Analyses were performed in gated CD4 and CD8 T cells. **e**, **f** BMDMs were cocultured in triplicate with T cells from *T. cruzi*-infected WT mice in the absence (closed circles) or presence (open squares) of the TAM receptor inhibitor Mer-Ig. **e** After 24 h, T cells were collected, counted, and labelled with anti-CD8 and 7-AAD for evaluation of 7-AAD^+^ dead cells. **f** BMDMs were treated with CFSE-labelled T cells for 1 h and then analysed for CD11b^+^ singlets as macrophages that internalized CFSE^+^ cells. Dotted control lines refer to **e** T cells cultured alone, and **f** macrophages cultured with medium only. The means and SEM of *n* = 3 technical replicates are represented. Significant differences between different macrophages are indicated for ^*^P < 0.05, ^**^P < 0.01, ^***^P < 0.001, and ^****^P < 0.0001, as analysed by ANOVA with Tukey’s posttest or by ANOVA followed by Bonferroni posttest of selected pairs of data (macrophages cocultured with T cells in the absence of treatment *versus* those treated with Mer-Ig). The results are representative of 3 independent experiments in **a**, **b**, and **c**. Panels **e** and **f** refer to 2 separate experiments.

Next, we employed cocultures with T cells from normal and infected mice to address differences in efferocytosis by WT and TAM-deficient macrophages, as schematically represented in Supplementary Fig. 3c. After 48 h, the recovery of T cells from cocultures was assessed by counting nonadherent cells. By using efferocytosis sufficient WT macrophages in cocultures, the recovery of T cells isolated from infected mice was reduced by approximately 50% compared with naïve T-cell counterparts. In contrast, T-cell recovery was approximately 70 and 90% in cocultures with Axl- and Mer-deficient macrophages, respectively (Fig. 3a). Reduced T-cell counts in cocultures with T cells from infected mice seemed to correlate with the active removal of apoptotic cells by WT macrophages because culture in the absence of macrophages increased T-cell recovery (Fig. 3b). Moreover, the lack of Axl and Mer receptors resulted in progressive accumulation of T cells in cocultures. (Fig. 3b). Furthermore, increased numbers of dead CD4^+^ and CD8^+^ T cells were detected in cocultures with Axl- and Mer-deficient macrophages (Fig. 3c, d). To evaluate the proportional role of Axl and Mer in efferocytosis, we employed the TAM receptor inhibitor Mer-Ig for 24 h in parallel with analyses of TAM-defective macrophages. In cocultures between T cells from infected mice and WT macrophages, treatment with the TAM receptor inhibitor increased both the percentages and absolute numbers of dead CD8 T cells near the macrophage-free control levels (Fig. 3e). In addition, the TAM receptor inhibitor further increased the recovery of dead CD8 T cells in the presence of Mer-defective macrophages, indicating the involvement of a second TAM receptor (Fig. 3e). These results indicate that both Axl and Mer contribute to efferocytosis and play a major role in the removal of dead cells by WT macrophages. Further analyses of the internalization of CFSE^+^ apoptotic T cells in 1 h-short assays (Fig. 3f and Supplementary Fig. 4) confirmed that the absence of Axl or Mer, as well as treatment with the TAM receptor inhibitor, reduced efferocytosis. Altogether, these findings are consistent with the hypothesis that Mer and Axl play a major role in the efferocytosis of T cells from *T. cruzi*-infected mice, although at different efficiencies.

### Axl, but not Mer, suppresses T-cell induction of M1 effector macrophages

**Fig. 4:**
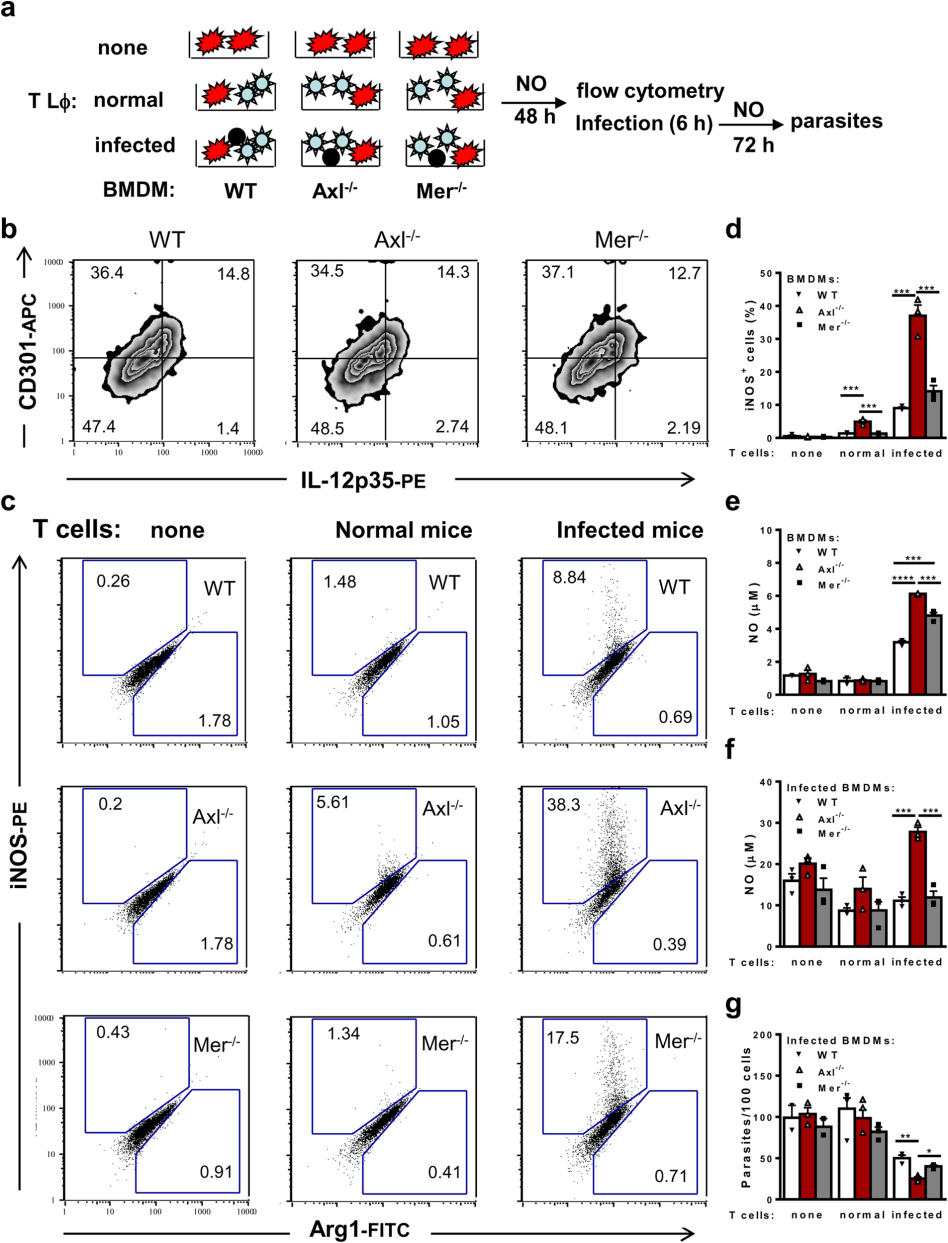
Axl-receptor defective macrophages express improved induction of M1 responses. **a** BMDMs from WT (closed triangles), Axl^−/−^ (open triangles) and Mer^−/−^ (closed squares) mice were cultured in triplicate in medium only or with T cells from normal or infected (15 dpi) WT mice. After 48 h, culture supernatants and nonadherent cells were removed, and adherent cells were stained for flow cytometry. In parallel cultures, macrophages were infected with *T. cruzi* metacyclic forms (multiplicity of infection, MOI of 8:1), washed, and cultured for an additional 72 h period. **b**, **c** In the plots, F4/80^+^ cells were gated and then analysed for **b** surface CD301 and intracellular IL-12p35 in macrophages cultured with medium only; **c** intracellular iNOS and Arg1 expression in macrophages treated as indicated. **d** Graphs depict iNOS expression. **e**, **f** Supernatants were evaluated for NO production. **g** Intracellular parasites were counted by light microscopy. The results are expressed as the means and SEM of *n* = 3 technical replicates. Significant differences between different macrophages are indicated for ^*^P < 0.05, ^**^P < 0.01, ^***^P < 0.001, and ^****^P < 0.0001, as analysed by ANOVA with Tukey’s posttest. The results are representative of 3 independent experiments. In **g**, the data represent 2 independent experiments.

Next, we employed cocultures to assess the phenotypes of F4/80^+^ BMDMs from WT, Axl^−/−^, and Mer^−/−^ mice (Fig. 4a). In the absence of T cells, BMDMs from all strains contained a predominant population of CD301 single^+^ (M2) cells as well as CD301^+^IL-12^+^ (M2-M1 intermediate) cells (Fig. 4b), but they did not express iNOS (Fig. 4c). In addition, WT, Axl^−/−^, and Mer^−/−^ macrophages infected equally, as assessed 3 h after infection with 5 parasite/macrophage ratios (WT: 16 ± 3; Axl^−/−^: 15 ± 3; Mer^−/−^: 13 ± 3 parasites/100 macrophages). These results indicate that the absence of Axl or Mer does not affect per se the macrophage phenotype in synchronized BMDMs. Next, we investigated how the absence of each TAM receptor would affect macrophage functional phenotypes (Fig. 4 and Supplementary Fig. 5). As expected, coculture with in vivo-primed T cells from infected but not normal mice induced iNOS expression (Fig. 4c, d) and NO production (Fig. 4e) by WT macrophages even in the absence of infection. Moreover, macrophages from Axl-deficient mice further upregulated iNOS expression and NO production in comparison with WT and Mer^−/−^ macrophages (Fig. 4c–e). After 48 h in parallel cultures (Fig. 4a), the macrophages were washed and infected to reveal the ability of macrophages to produce NO and deal with intracellular infection. Compared with noninfected macrophages in Fig. 4e, in vitro infection stimulated increased NO production in each macrophage group (Fig. 4f). Nonetheless, parasites were not eliminated by macrophages previously treated with medium only or with normal T cells (Fig. 4g). In contrast, macrophages that had been exposed to T cells from infected mice performed better for parasite elimination (Fig. 4g). However, infected Axl^−/−^ but not Mer^−/−^ macrophages had even improved NO responses and better control of parasite infection (Fig. 4f, g). In the presence of T cells from infected mice, Axl-defective macrophages also downregulated the CD301 (MGL) M2 marker coupled with increased expression of M1 hallmarks, such as IL12p35 and CXCL9 (Supplementary Fig. 5c–f). Nonetheless, we did not find any consistent difference in the production of IL-10 or IFN-γ in cocultures contained WT, Axl^−/−^ or Mer^−/−^ macrophages (Supplementary Fig. 5g–i). Overall, whereas Mer plays a predominant role in the removal of apoptotic cells, Axl seems to suppress T-cell-induced M1 effector macrophages upon detection of apoptotic T cells from infected mice.

### Axl-deficient but not Mer-deficient mice are less susceptible to experimental Chagas disease

**Fig. 5:**
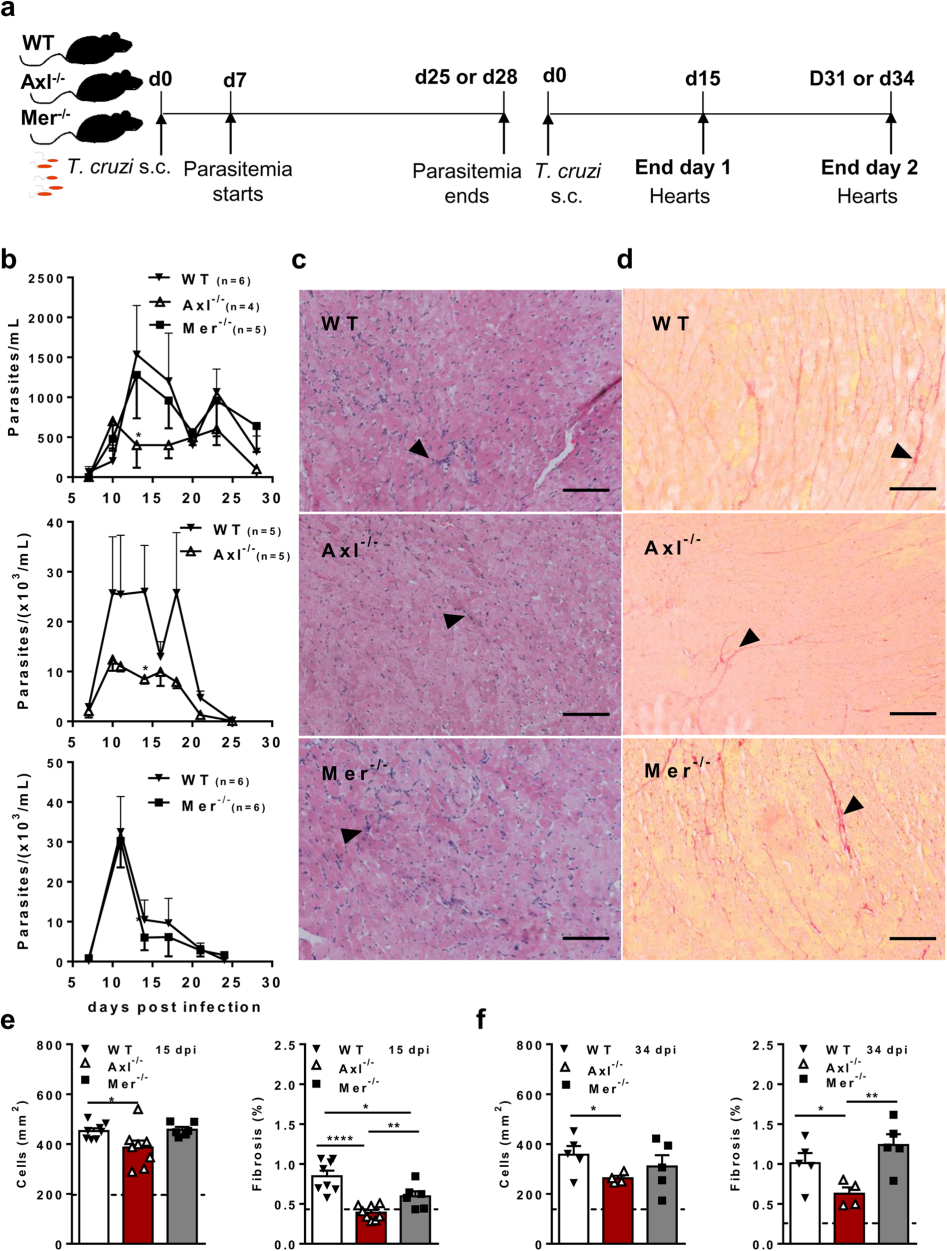
Axl receptor engagement impairs early control of *T. cruzi* infection. **a** Male WT (closed triangles), Axl^−/−^ (open triangles) and Mer^−/−^ (closed squares) mice were infected sc with *T. cruzi*. **b** Parasitemia in WT, Axl^−/−^ and Mer^−/−^ mice was followed throughout acute infection in separate experiments. The number (*n*) per mouse group, the means, and SEM are indicated in the figures. Significant differences between WT and Axl^−/−^ mice are indicated for ^*^P < 0.05 as analysed by unpaired *t*-test in normalized data. **c**, **d** Representative micrographs of H&E- and picrosirius red-stained heart sections from infected WT, Axl^−/−^ and Mer^−/−^ mice (34 dpi). Bars: 100 µM. Arrowheads point to areas with **c** inflammatory cells and **d** fibrosis. **e**, **f** Graphs represent cell infiltrates as expressed in cells/mm^2^ and collagen deposition (area of fibrosis) in the hearts obtained at **e** 15 dpi and **f** 34 dpi. The results represent **e** 1 and **f** 2 independent experiments. Dashed lines represent basal levels in naïve WT mice. The results are expressed as the means and SEM for each experimental group; **e** WT (*n* = 8), Axl^−/−^ (*n* = 8), and Mer^−/−^ (*n* = 6) infected mice; **f** WT (*n* = 5), Axl^−/−^ (*n* = 4), and Mer^−/−^ (*n* = 5) infected mice. Significant differences in unpaired Student’s *t*-tests are indicated for ^*^P < 0.05, ^**^P < 0.01, and ^****^P < 0.0001.

To investigate how each TAM receptor affects host immune responses to *T. cruzi*, parasitemia was followed in infected WT, Axl^−/−^, and Mer^−/−^ mice (Fig. 5a). Here, we show that Axl-deficient but not Mer-deficient mice expressed reduced peak parasitemia during *T. cruzi* infection (Fig. 5b). Importantly, the hearts from infected Axl^−/−^ but not Mer^−/−^ mice presented reduced inflammation and fibrosis throughout acute infection, both at 15 dpi and at 34 dpi (Fig. 5c–f and Supplementary Fig. 6). At 15 dpi, we also detected inflammatory cells in close vicinity with parasite nests in haematoxylin eosin (HE)-stained micrographs (Supplementary Fig. 6c). Furthermore, the numbers of iNOS^+^ cells increased within inflammatory infiltrates from infected Axl^−/−^ mice compared with infected WT and Mer-defective mice (Supplementary Fig. 7). Therefore, Axl might disrupt parasite control by reducing M1-effector responses, thereby contributing to infection-driven inflammatory responses in the heart.

### Accumulation of apoptotic T cells in *T. cruzi*-infected Axl^−/−^ mice

**Fig. 6:**
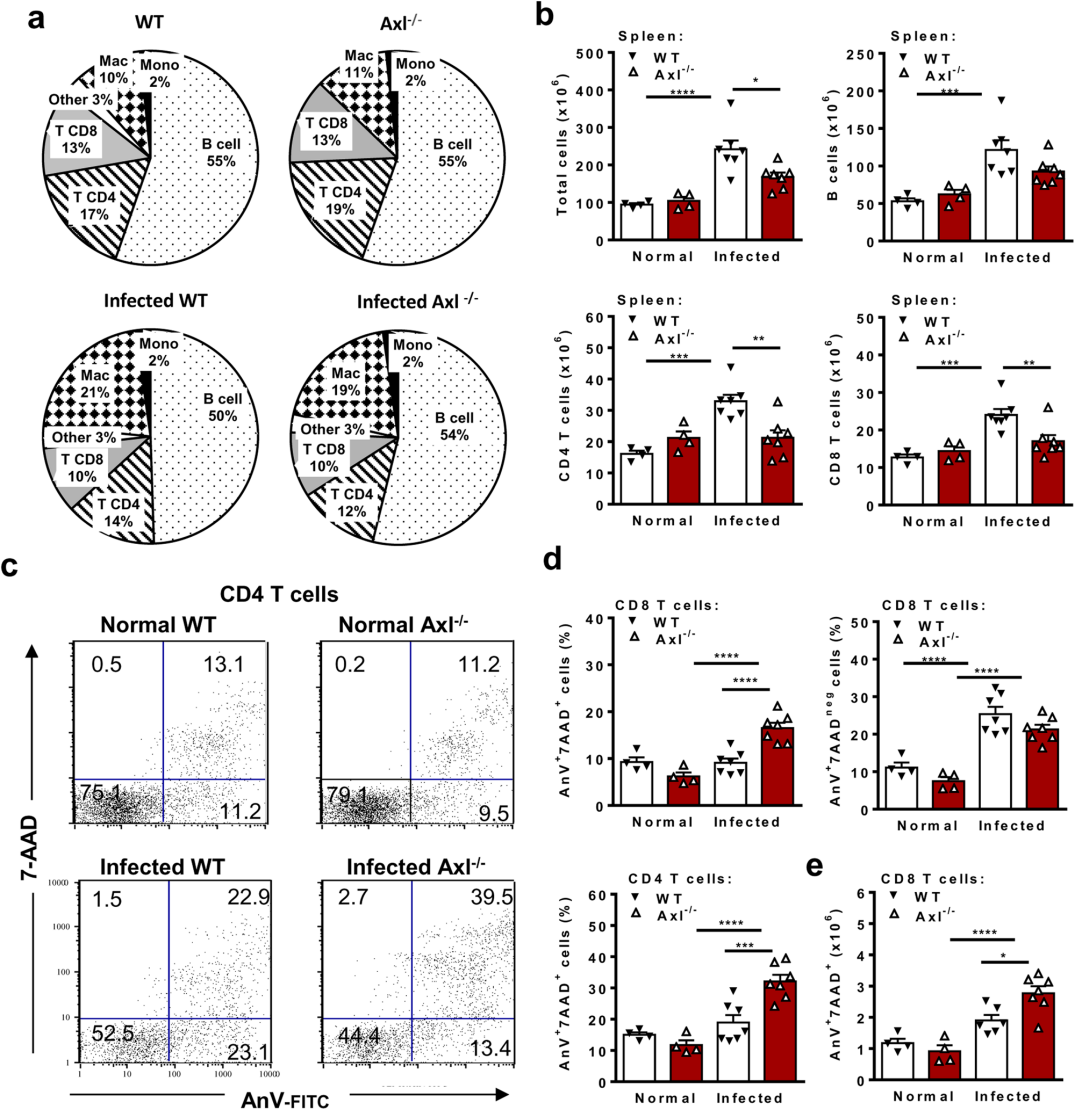
The Axl receptor promotes efferocytosis of proapoptotic T cells during acute infection. Male Axl^−/−^ (open triangles) and WT (closed triangles) mice were infected sc with *T. cruzi* parasites. Normal Axl^−/−^ (open triangles) and WT (closed triangles) mice were used as controls. At 2 wpi, splenocytes were obtained and analysed by flow cytometry. **a** Each cell population (%) is represented as the mean for each experimental group. **b** Graphs depict the absolute numbers of total splenocytes, B cells, CD4 T cells, and CD8 T cells. **c** Representative plots show late (7-AAD^+^AnV^+^) and early (AnV^+^7-AAD^neg^) apoptosis for CD4 T cells in WT and Axl^−/−^ mice. **d** The percentages of early and late apoptotic T cells are represented for each mouse. **e** Accumulation of late-apoptotic CD8 T cells in infected Axl^−/−^ mice. The results are expressed as the means and SEM of *n* = 4 normal WT and Axl^−/−^ mice and *n* = 7 infected WT and Axl^−/−^ mice for each experimental group. Significant differences, as analysed by ANOVA followed by Bonferroni posttest (noninfected *versus* infected mice; Axl^−/−^ *versus* WT mice), are indicated for *P < 0.05, ^**^P < 0.01, ^***^P < 0.001, and ^****^P < 0.0001. All experimental data were analysed except for the outlier in **e** removed from the infected WT group after Grubbs’ test.

**Fig. 7:**
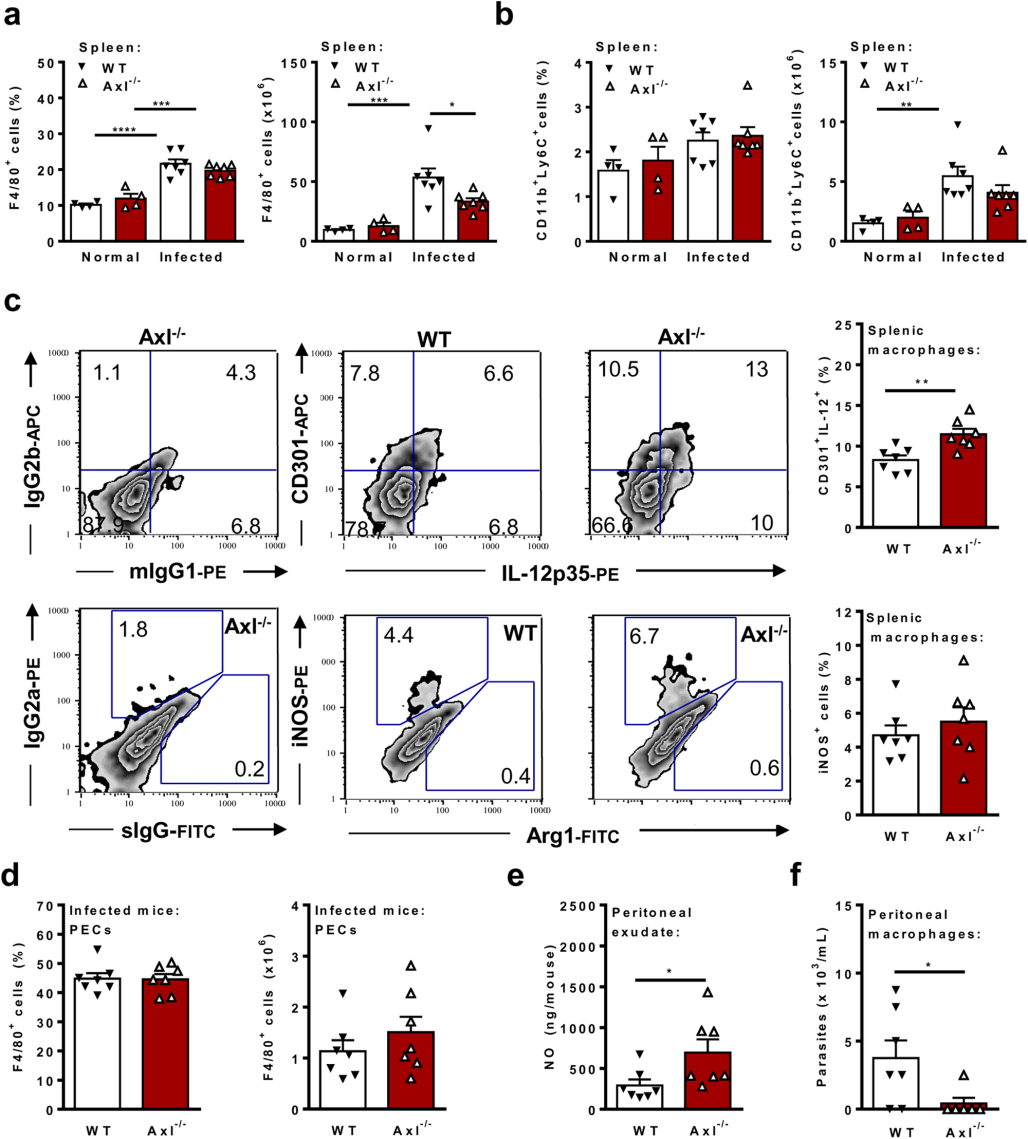
Defective Axl engagement upregulates macrophage-mediated immunity. Male Axl^−/−^ (open triangle) and WT (closed triangle) mice were infected sc with *T. cruzi* parasites. Normal Axl^−/−^ and WT mice were used as controls. At 14 dpi, splenocytes and peritoneal exudate cells were analysed by flow cytometry. **a**, **b** Percentages and absolute numbers of F4/80^+^ macrophages and CD11b^+^Ly6C^+^ monocytes from the spleens are represented for each mouse. **c** Representative plots show the expression of extracellular CD301 and intracellular IL-12p35, iNOS, and Arg1 in F4/80^+^ splenocytes. The graphs depict the percentages of CD301^+^IL12p35^+^ cells and iNOS^+^ macrophages. **d** Percentages and absolute numbers of F4/80^+^ peritoneal macrophages. **e** NO production in infected WT and Axl^−*/*−^ mice, as evaluated in 5 mL-diluted peritoneal exudates. **f** Parasite load of in vitro-infected peritoneal macrophages. The means and SEM of *n* = 4 normal WT mice, *n* = 4 Axl^−/−^ normal mice, *n* = 7 infected WT mice, and *n* = 7 infected Axl^−/−^ are shown. Significant differences, as analysed (in **a** and **b**) by ANOVA followed by Bonferroni posttest (noninfected *versus* infected mice; Axl^−/−^ *versus* WT mice) or (in **c**, **d**, **e**, and **f**) by unpaired Student’s *t*-tests (infected Axl^−/−^ *versus* infected WT mice) are indicated as for ^*^P < 0.05, ^**^P < 0.01, ^***^P < 0.001, and ^****^P < 0.0001.

For ex vivo analyses of systemic immune responses, we infected WT and Axl^−/−^ mice, and as expected, parasitemia increased from 10 to 13 dpi in WT but not Axl^−/−^ mice (Supplementary Fig. 8a, b). Next, we evaluated splenocytes from WT and Axl^−/−^ mice at 14 dpi for lymphocyte (Fig. 6) and macrophage (Fig. 7) subpopulations. The frequencies of each major splenic subpopulation were similar between WT and Axl^−/−^ mice, either prior to or after infection (Fig. 6a). Interestingly, the ratio between T cells and F4/80^+^ macrophages decreased from 3:1 before infection to approximately 1:1 at 14 dpi (Fig. 6a). Nonetheless, the absolute numbers of total splenocytes and B and T cells increased in WT but not Axl^−/−^ mice upon infection (Fig. 6b). Therefore, we observed a reduced expansion of T cells in infected Axl^−/−^ mice compared with WT mice (Fig. 6b). Next, we addressed the role of Axl in efferocytosis by evaluating T-cell apoptosis during infection. Strikingly, whereas T-cell apoptosis ratios increased after infection in WT and Axl^−/−^ mice (Fig. 6c), the percentages of late (7-AAD^+^AnV^+^ cells), but not early (7-AAD^neg^AnV^+^ cells), apoptotic cells increased only in CD4 and CD8 T cells from infected Axl^−/−^ mice (Fig. 6c, d). Furthermore, the absolute numbers of CD8 T cells that underwent late apoptosis also increased during infection in Axl^−/−^ mice (Fig. 6e). Altogether, these results are consistent with the idea that defective Axl-mediated efferocytosis leads to either failed or delayed clearance of apoptotic T cells, which accumulated as late apoptotic cells.

### Axl^−/−^ mice express improved macrophage-mediated immunity to *T. cruzi* infection

To address macrophage effector phenotypes upon infection, we evaluated splenic F4/80^+^ macrophages and CD11b^+^Ly6C^+^ monocytes. The frequencies of macrophages, but not monocytes, increased during *T. cruzi* infection in both WT and Axl^−/−^ mice, whereas the absolute numbers of macrophages and monocytes increased only in WT mice (Fig. 7a, b). In contrast, we observed increased expression of effector phenotypes in macrophages from infected Axl^−/−^ mice (Fig. 7c). We found increased proportions of splenic F4/80^+^ macrophages expressing both CD301 and IL-12p35 (Fig. 7c), as well as increased expression of IL-12p35 and iNOS by CD11b^+^Ly6C^+^ monocytes from infected Axl^−/−^ mice (Supplementary Fig. 8d). We also evaluated peritoneal immune responses (Figs. 7d–f) during infection (14 dpi). There was no significant difference in the frequencies or absolute numbers of peritoneal macrophages in infected Axl^−/−^ and WT mice (Fig. 7d). Remarkably, infected Axl^−/−^ mice expressed increased NO production in peritoneal exudates compared to infected WT mice (Fig. 7e). Moreover, peritoneal macrophages from infected WT but not Axl^−/−^ mice were more susceptible to infection in vitro (Fig. 7f). Considering that there are equivalent proportions of F4/80^int^ immature macrophages, IL-12^+^CD301^+^ cells, and iNOS^+^ macrophages in both genotypes (Supplementary Fig. 9a) in face of increased proinflammatory cytokine responses in infected WT mice (Supplementary Fig. 9b), Axl^−/−^ peritoneal macrophages showed an intrinsic improved effector response (Fig. 7e, f).

### Reduced efferocytosis in macrophages from infected Axl^−/−^ mice

**Fig. 8:**
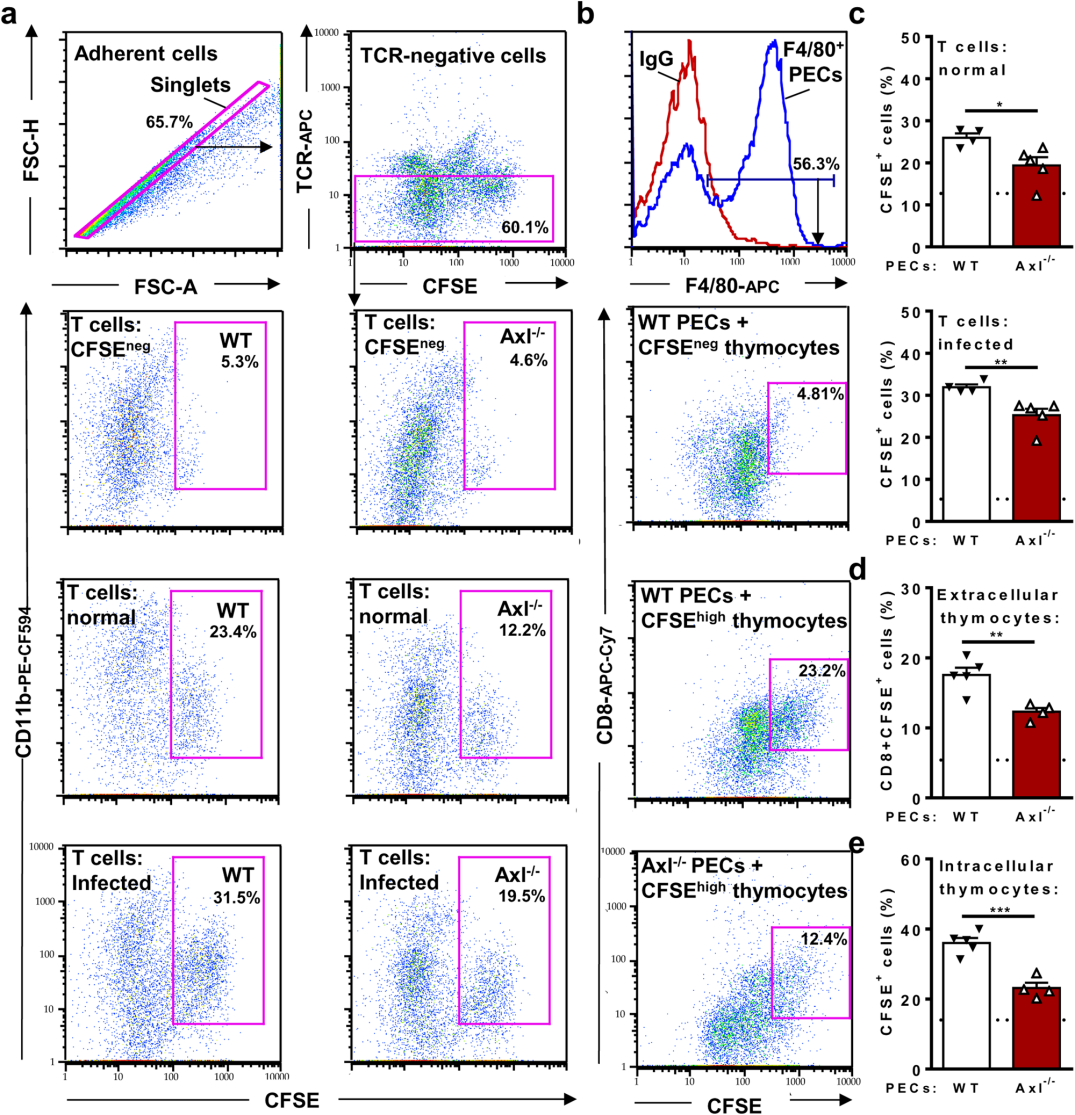
Defective efferocytosis by peritoneal macrophages from Axl^−/−^ infected mice. PECs from infected Axl^−/−^ (open triangles) and infected WT (closed triangles) mice were treated with CFSE-labelled or CFSE-negative T cells or thymocytes for 1 h. **a**, **b** Adherent PECs were then analysed by flow cytometry for the presence of **a** intracellular or **b** extracellular CFSE^+^ cells. **a, c** PECs treated with CFSE-labelled T cells were washed and then stained with anti-CD11b and anti-TCRβ mAbs. For evaluation of intracellular CFSE^+^ cells, doublets and TCR^+^ cells were gated out and excluded from analysis. To define CD11b^+^CFSE^+^ macrophages, gates were based on macrophages treated with CFSE-negative T cells, employed as negative controls. **c** Graphs show the percentages of intracellular T cells in PECs from infected WT (*n* = 4) and Axl^−/−^ (*n* = 5) mice. **b, d** For analysis of tethering, a more homogenous population of CFSE-labelled apoptotic thymocytes was used in the assays. PECS were washed and stained with anti-F4/80 and anti-CD8. Then, gated F4/80^+^ cells were analysed for extracellular CD8^+^CFSE^hi^ thymocytes. Gates were based on macrophages treated with CFSE-negative thymocytes. **d** Graphs depict the percentages of PECs from infected WT (*n* = 5) and Axl^−/−^ (*n* = 4) mice bound to extracellular (CD8^+^) thymocytes. **e** F4/80^+^ macrophages containing intracellular thymocytes. Selection of singlets followed by gating of F4/80^+^ macrophages was used to identify macrophages that internalized CFSE^+^ cells. Each symbol represents the average of duplicates/triplicate determinations for each mouse. Dotted lines stand for WT PECs treated with CFSE-negative cells. Means and SEM are represented for each mouse group. Significant differences, as analysed by unpaired Student’s *t* tests, are indicated as for ^*^P < 0.05, ^**^P < 0.01, and ^***^P < 0.001.

To directly assess efferocytosis, peritoneal macrophages from infected mice and splenic T cells were separately cultured for 24 h. Then, macrophages were treated with CFSE-labelled (dead) cells for 1 h to allow their binding and internalization (Supplementary Fig. 10). To evaluate macrophages that phagocytosed CFSE^+^ T cells, CD11b^+^CFSE^+^ cells were analysed after gate exclusion of doublets and TCR^+^ cells. Compared with infected WT mice, peritoneal macrophages from Axl^−/−^ mice showed defective efferocytosis of T cells from normal or infected mice (Fig. 8a, c). We also assessed tethering, an initial stage of efferocytosis, by using a homogenous population of apoptotic CFSE^+^ thymocytes (Supplementary Fig. 10d). Compared with F4/80^+^ peritoneal macrophages from infected WT mice, Axl-deficient macrophages had reduced binding to extracellular CD8^+^CFSE^+^ thymocytes (Fig. 8b, d) as well as defective internalization of CFSE^+^ thymocytes (Fig. 8e).

Altogether, these results indicate that Axl induces efferocytosis and suppresses macrophage-mediated immunity during acute *T. cruzi* infection, resulting in increased parasitemia, overall immune responses, and heart pathology.

## Discussion

Our previous findings indicate that inhibition of lymphocyte apoptosis improves immune responses mediated by T cells and macrophages in *T. cruzi* infection^13,22–24,26^: i) directly by rescuing effector T cells or delaying their death, and/or ii) indirectly by preventing or retarding the phagocytosis of apoptotic cells by macrophages. Whereas both scenarios provide reasonable explanations for the effects of apoptosis inhibition^26^, how efferocytosis alone affects macrophage-mediated immunity to *T. cruzi* infection requires the direct assessment of macrophage receptors involved in efferocytosis in vivo. Here, we show that genetic ablation of Axl receptor reduces efferocytosis and is sufficient to upregulate M1 macrophage immunity, reduce peak parasitemia, and prevent heart pathology in experimental Chagas disease.

Multiple pairs of ‘eat me’ signals and receptors might be involved in macrophage-mediated efferocytosis in infectious diseases^30^. We previously investigated the role of the αvβ3 integrin in vitro and found that: i) peritoneal macrophages from *T. cruzi*-infected mice phagocytosed apoptotic naïve T cells in an integrin-dependent fashion, and ii) αvβ3-mediated efferocytosis exacerbated *T. cruzi* infection by inducing PGE-2, TGF-β, ornithine decarboxylase activity and polyamine production^13^. Here, we show that Axl receptor also plays a role in both tethering and internalization of apoptotic cells by peritoneal macrophages from infected mice.

By addressing the role of TAM receptors, we found that both Axl and Mer promote BMDM-mediated efferocytosis of proapoptotic T cells from *T. cruzi*-infected mice. Although the sources of macrophages and apoptotic T cells are distinct in this and the previous experimental model, it is conceivable that a few or multiple receptors, such as αvβ3, Axl, Mer, and other putative receptors, such as TIM4^41^, work in concert in efferocytosis, as previously suggested^42–44^. Moreover, upon binding to apoptotic cell ligands, receptors might cluster together in an efferocytosis-induced molecular platform dedicated to tethering, phagocytosis, and inhibitory signalling response to apoptotic cells. Nonetheless, the molecular components in this efferocytosis synaptic complex and their respective signalling pathways might vary considerably depending on the apoptotic cell stage, macrophage type, and immune context. Here, we show that the TAM receptor inhibitor Mer-Ig blocked efferocytosis and improved M1 responses to immune T cells from infected mice. Moreover, treatment with Mer-Ig upregulated M1 responses in cocultured BMDMs from *T. cruzi*-resistant (Th1-prone) B6 and *T. cruzi*-susceptible (Th2-prone) BALB/c mice. Whereas susceptibility and resistance to intracellular protozoan parasites are related to various features of both innate and adaptative immunity in these particular mouse strains^3,45^, efferocytosis-mediated suppression of macrophage responses may also promote parasite infection. For instance, our results that show improved M1 responses with the use of the TAM inhibitor corroborate the role of TAM receptors in *L. major* infection, where Axl^−/−^Mer^−/−^ (double KO) mice showed increased innate responses of dermal macrophages^39^. Therefore, TAM receptor inhibitors represent potential for immune stimulation^33^ in infectious diseases, which rely on macrophages as immune and host cells. Nevertheless, caution should be advised for the advent of potential side effects upon TAM inhibition, such as defective cross-presentation by DCs or otherwise increased inflammatory responses and pathology secondary to the blockade of efferocytosis-dependent regulatory circuits^39,46–49^. The identification of selective roles of single TAM receptors might reduce the side effects of blocking them altogether.

To address the role of individual TAM receptors, we used synchronized BMDMs from WT, Axl- and Mer-defective mice cocultured with T cells from *T. cruzi*-infected mice. Whereas Mer and Axl are expressed and predominate, respectively, in homeostasis and inflammation^36,37^, coculture with infection-primed T cells upregulated the frequency of macrophages coexpressing Mer and Axl. Overall, macrophage expression and efferocytosis mediated by Mer predominated over Axl in BMDMs treated with T cells from *T. cruzi*-infected mice. Nonetheless, we identified Axl as a major regulator of effector macrophage responses to immune T cells. Axl^−/−^ macrophages had the highest expression of M1 responses, such as iNOS, NO, IL-12, and the M1-chemokine CXCL9, but reduced M2 (CD301) marker expression and better control of parasite infection. Our results corroborate that efferocytosis by Axl and Mer could induce qualitatively or quantitatively distinct signalling pathways, transcriptional repressors^50^ and responses^36^ in addition to the differential use of the bridge molecules Gas6 and ProS^51^. Axl and Mer might also associate with different molecular partners^41,47^ in the efferocytosis synaptic complex to regulate immune responses through their intrinsic signalling pathways. TAM receptors have been shown to express intrinsic tyrosine kinase activity, which diverts associated IFNAR responses^35,48,52^ towards SOCS1 and SOCS3 induction. Whether distinct molecular partners for Mer and Axl differentially control the induction of SOCS 1 and 3 in any condition remains uncertain. Remarkably, SOCS1 influences macrophage function not only by shifting to the M2 phenotype but also by restricting M1 proinflammatory cytokine and NO responses^53^. The identification of critical Axl signaling pathways and/or suppressive cytokine responses that control macrophage phenotypes deserves further investigation.

To investigate the role of each TAM receptor Axl and Mer in vivo, we employed an experimental Chagas disease model. Strikingly, parasitemia did not peak in Axl^−/−^ mice, although *T. cruzi* could be detected in low parasitemia throughout acute infection. In contrast, infection followed a similar course in WT and Mer^−/−^ mice. Therefore, inhibition of Axl but not the Mer receptor has therapeutic potential to reduce parasite infection. We hypothesized that Axl downregulates macrophage-mediated immunity to *T. cruzi* infection, and to address this issue, we analysed the immune responses by the time Axl^−/−^ mice showed reduced peak parasitemia. We did not find differences in the frequencies of different splenocytes between Axl^−/−^ and WT mice, whereas the absolute T-cell numbers were increased only in WT mice at peak parasitemia. Nevertheless, accumulation of apoptotic T cells at a later apoptosis stage in infected Axl^−/−^ mice stands as indirect evidence of defective efferocytosis. We have shown increased frequencies of macrophages in WT and Axl^−/−^ mice at the time that parasitemia peaks in WT mice. Moreover, both macrophages and monocytes from the spleens, as well as inflammatory cells in the hearts of Axl mice expressed improved M1-like responses during *T. cruzi* infection. Furthermore, higher NO production was detected in peritoneal exudates, and peritoneal macrophages from infected Axl^−/−^ mice controlled parasite infection better in vitro. For direct assessment of efferocytosis, we detected tethering and internalization of CFSE^+^ apoptotic cells by peritoneal macrophages. Strikingly, peritoneal macrophages from infected Axl^−/−^ mice showed defective efferocytosis compared with WT macrophages. Altogether, these results indicate that Axl-mediated efferocytosis by macrophages might downmodulate immunity to *T. cruzi* infection, leading to peak parasitemia. In addition, macrophage activation could also occur because of the accumulation of late apoptotic cells, which can undergo secondary necrosis and release damage-associated molecular patterns (DAMPs), as previously suggested^46,54^. Nonetheless, our in vitro results do not support this nonexcluding hypothesis. Although dead T cells accumulated predominantly in Mer^−/−^ macrophage cocultures, Axl^−/−^ but not Mer^−/−^ macrophages expressed the highest M1 responses. Paradoxically, excessive accumulation of apoptotic T cells in coculture with Mer-deficient macrophages might eventually increase suppression through other inhibitory receptors, including Axl.

The outcome of complex interactions between macrophages and (apoptotic and effector) T cells will dictate macrophage ability to deal with parasite infection. Before adaptive immune responses achieve parasite control, macrophages seem to promote infection in an Axl-dependent fashion that culminates in failure to develop a full M1 effector phenotype. Our results do not support the hypothesis of early control of parasitemia by improved T-cell help to macrophages in Axl^−/−^ mice, owing to unrestricted dendritic cell APC functions, as previously suggested in *L. major*-infected Axl^−/−^Mer^−/−^ mice^39^ or in mice treated with anti-Axl^55^. Otherwise, *T. cruzi*-infected Axl^−/−^ mice expressed reduced peritoneal cytokine responses, as well as decreased numbers of CD4 and CD8 T cells in the spleen, compared with WT mice (Fig. 6 and Supplementary Fig. 9). These results seem to correlate better with lower parasitemia and reduced parasite-driven immune responses in infected Axl^−/−^ mice, secondary to unleashed M1 responses and increased parasite control. Overall, our data are more compatible with an intrinsic macrophage dysfunction in response to complex environmental stimuli composed of cytokines and apoptotic cells in an Axl-dependent manner. Although the in vitro model recapitulated the results obtained with infected mice, we cannot rule out potential efferocytosis-independent or even macrophage-independent^56^ Axl effects. Further investigation will be necessary to address efferocytosis-independent Axl effects or whether TAM receptors play any role on the transfer of *T. cruzi* parasites from infected apoptotic cells to macrophages.

Finally, irrespective of the fact that *T. cruzi* can infect multiple cell types, macrophages might play a key role in controlling *T. cruzi* infection, as previously evidenced by increased parasitemia, tissue parasitism, and mortality in macrophage-depleted mice and rats^57,58^. Because macrophages insensitive to IFN-γ (MIIG) are unable to produce NO or kill *T. cruzi* parasites in vitro^10^, MIIG mice are highly susceptible to *T. cruzi* infection with increased parasitemia and mortality associated with high tissue parasitism and inflammation^10^. Likewise, M2 macrophages have been suggested to be more susceptible to *T. cruzi* infection^12^, and the shift of cardiac M2 to the M1 phenotype correlates with the control of tissue parasitism and better myocardial functioning^11^.

Here, we show that *T. cruzi*-infected Axl^−/−^ mice had reduced inflammation and fibrosis in their hearts compared to WT or Mer^−/−^ mice, possibly owing to an improved M1 shift and early control of parasitemia. By employing the caspase-inhibitor zVAD^24^ and anti-FasL^23^ during infection, we observed upregulated T-cell responses secondary to apoptosis inhibition and reduced parasitemia. Nonetheless, these pharmacological approaches were unable to prevent and can increase inflammation in the heart^24,28^. In contrast, infected Axl^−/−^ mice controlled better parasitemia without upregulating potentially pathological T-cell responses and ameliorated heart disease. Because the interplay between parasite infection and T-cell responses underlies pathogenesis in Chagas disease^2,3^, the induction of improved M1 responses without further upregulation of effector T cells might correlate with better disease outcomes in Axl-defective mice.

Overall, strategies that improve early macrophage activation upon infection or that modulate efferocytosis may help to control *T. cruzi* infection, opening new therapeutic avenues for immunoregulation in Chagas disease. Importantly, increased T-cell apoptosis correlated with heart failure in human chronic Chagas disease^59,60^. Other intracellular pathogens might conceivably take advantage of Axl or efferocytosis by macrophages to inhibit immune responses. Whether the therapeutic targeting of Axl-mediated efferocytosis will provide new treatments or vaccines for Chagas disease and other related infections deserves further investigation.

## Methods

### Mice and *T. cruzi* infection

C57BL/6 (B6) and BALB/c mice were obtained from the Oswaldo Cruz Foundation (FIOCRUZ, Rio de Janeiro, Brazil). *Axl*^*-/-*^ (Jackson Lab #011121) and *Mertk*^*-/-*^ (#011122) mouse strains^61,62^ [which were backcrossed for at least 6 and 9 generations to the C57BL/6 background]^34,63^ were donated by Brian Kelsall (National Institutes of Health, USA). WT (B6) mice were used as controls. All animals were transferred to and housed in ventilated microisolators in the animal facility at the Federal University of Rio de Janeiro (UFRJ). The animal study was reviewed and approved by the Ethics Committee for Use of Animals at the Federal University of Rio de Janeiro (CEUA-UFRJ). All experiments were conducted as in protocols 078/16 and A22/19-078-16, according to national and institutional regulations that comprise international standards. Male mice (aged 6–9 weeks) were infected subcutaneously (sc) with 2 × 10^5^ metacyclic trypomastigotes of the *Dm28c Trypanosoma cruzi* clone obtained by chemically induced metacyclogenesis in triatomine artificial urine-proline medium^64^. Parasitemia was determined at the indicated days post infection (dpi), and parasites were counted either in a Neubauer chamber or in 5 µL of undiluted blood smears examined on slides under glass coverslips by light microscopy. Experiments were conducted either during or at the end of the acute phase, and age-matched mice were used as naïve controls. Hearts were removed at 15 dpi or at the end of the acute phase (31 or 34 dpi). As a source of T cells for macrophage coculture, male BALB/c or WT (B6) mice were infected intraperitoneally (ip) with 2 × 10^5^ metacyclic trypomastigotes, and the spleens were removed at peak parasitemia (14–18 dpi).

### BMDM differentiation

Tibiae and femurs were removed from male or female mice (5–8 weeks) and washed with Hanks’ balanced salt solution (HBSS) (Gibco BRL, South America) plus 10% foetal bovine serum (FBS, Gibco). Cells were collected, washed, and cultured at 5 × 10^5^/10 mL in Petri dishes (90 × 20 mm^2^). Cultures were maintained for 7 days in Roswell Park Memorial Institute (RPMI) medium supplemented with 2 mM glutamine, 5 ×10^5^M 2-ME, 10 μg/mL gentamicin, 1 mM sodium pyruvate, 0.1 mM minimum essential medium (MEM) nonessential amino acids (all from Gibco), and 10% FBS. Cultures were maintained at 37 °C in a 7% CO2 humid atmosphere and treated twice with 30% L929 cell culture supernatant as a macrophage colony-stimulating factor (M-CSF) source. After 7 days, the cells were collected, washed, and cultured in medium only or cocultured with T cells, as explained below.

### Infection in macrophages and parasite load

Peritoneal exudate cells (PECs) from mice infected with *T. cruzi* were cultured in triplicate at 2 × 10^5^ cells/well (0.5 mL) in 48-well vessels for 2 h at 37 °C and then washed for the removal of nonadherent cells. Adherent macrophages were infected overnight at a metacyclic trypomastigote/macrophage ratio of 10:1, washed, and maintained in Dulbecco’s modified Eagle’s medium (DMEM, Invitrogen Life Technologies, Carlsbad, CA, USA) supplemented with 2 mM glutamine, 5 × 10^5^M 2-ME, 10 μg/mL gentamicin, 1 mM sodium pyruvate, and 0.1 mM MEM nonessential amino acids (culture medium) plus 10% FBS at 37 °C and 7% CO_2_ in a humid atmosphere. Trypomastigotes released from macrophages were counted in culture supernatants after 3 weeks of infection^65^. For the intracellular infection assay, BMDMs were seeded in triplicate at 5 × 10^5^ cells/well on glass coverslips placed in 24-well plates and cultured (1 mL/well) in medium only or with 5 × 10^5^ T cells from normal or infected mice. After 48 h, the supernatants were collected, T lymphocytes were washed out, and macrophages were then infected for 6 h at a metacyclic trypomastigote/macrophage ratio of 8:1. Extracellular parasites were washed out, and infected macrophages were further cultured for 3 days for nitric oxide and infection assays. For parasite load, coverslips were stained with a Panotico kit (Laborclin, Brazil), and parasites were counted within macrophages. The results are expressed as numbers of parasites/100 macrophages.

### T cells

Splenocytes from normal or *T. cruzi*-infected mice (14–18 dpi) were obtained and depleted of red blood cells by treatment with Tris-buffered ammonium chloride, followed by nylon wool filtration to obtain T-cell-enriched suspensions (75–85% of TCRβ^+^ cells).

### Cocultured macrophages and T cells

For cocultures, BMDMs were settled in triplicate at 1–1.5 × 10^6^ cells/well in 24-well vessels in medium only or in the presence of 2-3 × 10^6 ^T cells from normal or infected mice at a T-cell/macrophage ratio of 2:1 unless indicated otherwise. Cultures (1 mL/well) were maintained in supplemented DMEM plus 10% FBS at 37 °C in a 7% CO2 humid atmosphere for 48 h. Cocultures are parasite-free unless otherwise stated in the figures and figure legends. In some experiments, macrophages from B6 or BALB/c mice were pretreated for 30 min with 4 or 5 µg/mL MerTK-IgG1Fc chimeric protein (R&D Systems, Minneapolis, MN, USA) and then treated with medium only or with T cells from infected mice for 48 h. Supernatants were collected for cytokine and NO assays, and the same volume of medium was added back. Lymphocytes were counted (Z1 Coulter particle counter, Beckman Coulter Inc., CA, USA) and processed for the apoptosis assay. Macrophages were then collected from plates and stained for flow cytometry.

### Flow cytometry assays

Splenocytes, PECs or cultured cells were washed in FACS buffer (phosphate-buffered saline plus 0.02% sodium azide and 3% FBS), followed by incubation with normal mouse serum or anti-CD16/CD32 (BD Biosciences, Chicago, IL, USA) for Fc blocking before staining with fluorochrome labelled antibodies (Supplementary Table 1). Splenic lymphocytes were stained with anti-CD4, anti-CD8 (eBioscience, San Diego, CA, USA) or anti-CD19 (BD Biosciences) labelled with allophycocyanin. For macrophage and monocyte staining, the following mAbs were employed: fluorescein isothiocyanate (FITC)-labelled anti-F4/80 (eBioscience) or allophycocyanin labelled anti-F4/80 (Biolegend, San Diego, CA, USA), allophycocyanin-labelled anti-CD11b (eBioscience) or phycoerythrin (PE)-labelled anti-CD11b (BD Biosciences), and FITC-labelled anti-Ly6C (Biolegend) or allophycocyanin-labelled anti-Ly6C (clone HK1.4, eBioscience). For TAM receptor expression, PE-labelled anti-MerTK (eBioscience) and allophycocyanin-labelled anti-Axl were employed. For macrophage phenotypes, cells were stained with allophycocyanin-labelled anti-CD301 (MGL) mAb or control rat IgG2b mAb (Biolegend); for intracellular staining, cells were washed, permeabilized, fixed, and stained with PE-labelled anti-IL-12p35 or control murine IgG1 mAb (R&D Systems); PE-labelled anti-NOS2 (iNOS) or control rat IgG2a mAb (eBioscience); FITC-labelled anti-arginase-1 or control sheep IgG mAb (R&D Systems). For defining subsets, gates were based on the exclusion of background staining with labelled-isotype control mAbs. For apoptosis detection, cells were first stained with allophycocyanin-labelled anti-CD8 or anti-CD4, washed, and then treated with FITC-labelled Annexin V (BD Biosciences or R&D Systems) for 20 min at room temperature, according to the manufacturer; 7-AAD (eBioscience) was added just before flow cytometry. For the efferocytosis assay, the following reagents and mAbs were employed: carboxyfluorescein succinimidyl ester (CFSE, Invitrogen Molecular Probes, Eugene, OR, USA), allophycocyanin-labelled anti-TCRβ (clone H57-597, BD Biosciences), allophycocyanin-Cy7 anti-CD8 (BD Biosciences) or control rat IgG2a mAb (Biolegend), allophycocyanin-labelled anti-CD11b (eBioscience) or PE-C594-labelled anti-CD11b (BD Biosciences), allophycocyanin-labelled anti-F4/80 (Biolegend), and fixable viability dye eFluor 450 (live/dead reagent, eBioscience). Cells were acquired in FACS Calibur (BD) systems by using the Cell Quest program (BD) or were acquired in BD LSRFortessa with the BD FACSDiva program for efferocytosis assays. FlowJow software (TreeStar, Ashland, OR, USA) was used for the analyses.

### Efferocytosis assays

To directly assess efferocytosis, apoptotic thymocytes and T cells were first stained with CFSE and then used to feed BMDMs or peritoneal macrophages. To obtain apoptotic cells for these assays, T cells from naïve or infected B6 mice were first cultured for 24 h in DMEM complete medium at 2 × 10^6^ cells/mL in 24-well plates. Thymocytes from normal B6 thymuses were cultured at 2 × 10^6^/mL in supplemented RPMI medium (24-well plates) for 72 h to obtain apoptotic thymocytes^66^. T cells and apoptotic thymocytes were then labelled with 0.5 µM CFSE. BMDMs and peritoneal macrophages from *T. cruzi*-infected mice were cultured at 1 × 10^6^ cells/well for 24 h and then incubated with 5 × 10^5^ CFSE-labelled thymocytes or T cells. After 1 h, nonadherent cells were washed out, and macrophages were collected and stained with anti-CD8/anti-TCRβ and anti-CD11b/anti-F4/80 for flow cytometry analyses. After excluding doublets and TCRβ^+^ cells, CD11b^+^CFSE^+^ cells correspond to macrophages that have phagocytosed (intracellular) CFSE^+^ cells. Gates were set based on the analyses of macrophages cultured alone or with CFSE-negative cells. Otherwise, gated F4/80^+^ cells were analysed for CD8^+^CFSE^+^ events for the identification of extracellular thymocytes.

### Cytokine and chemokine assays

Supernatants from cultures or diluted peritoneal exudates were used for detection of cytokines (IL-12p70, TNF-α, IL-10, and IFN-γ) and chemokines (CXCL9 and CCL17) by sandwich enzyme linked immunosorbent assays (ELISA). For detection, pairs of specific mAbs (Peprotech, Brazil and R&D Systems) were used, one of which was labelled with biotin, and the assay was then developed with streptavidin-alkaline phosphatase (Invitrogen Life Technologies, Waltham, MA, USA) and p-nitrophenyl phosphate (PNPP substrate, Thermo Scientific Pierce, Waltham, MA, USA), according to the manufacturers. Alternatively, avidin horseradish peroxidase (HRP) (eBioscience) and 3,3’,5,5’-tetramethylbenzidine (TMB substrate, eBioscience) were used for detection on a plate spectrophotometer (SpectraMax M5, Molecular Devices, San Jose, CA, USA).

### Nitric oxide assay

Production of NO by macrophages was determined indirectly by the quantification of nitrites. Peritoneal exudates (obtained by peritoneal washing with 5 mL of culture medium) or culture supernatants were mixed with the Griess reagent (1% sulfanilamide, 0.1% naphthylethylenediamine dihydrochloride, 2% H_3_PO_4_; Sigma Chemical Co, St. Louis, MO, USA) at a 1:1 ratio. A standard dilution curve of sodium nitrite (NaNO_2_) was used, and the results were expressed as nitrites (μM) or as ng/mL for peritoneal exudates. The optical density was determined at 540 nm on a plate spectrophotometer (SpectraMax M5).

### Histopathological analyses

Hearts from normal and infected (at 15, 31 or 34 dpi) mice were collected, fixed in buffered 10% formalin, dehydrated, and embedded in paraffin blocks. Microtome sections were stained with haematoxylin eosin (HE) for microscopic analysis of cell infiltrates or with picrosirius red for determination of fibrosis. Images were digitalized with an AxioCam MR Rev3 (Carl Zeiss, Oberkochen, Germany) camera adapted to a Zeiss Axioimager D2 microscope. For each mouse heart, at least 9-10 randomly (and blindly) selected fields were digitalized and then analysed by using the National Institutes of Health (NIH) ImageJ 1.53k program for the number of infiltrating cells and the percentage of fibrosis area. Parasite nests were identified and captured in micrographs from the hearts collected at 15 dpi.

### Immunohistochemistry

For immunohistochemistry (IHC), heart paraffin sections were permeabilized and endogenous peroxidase was then inhibited with 3% hydrogen peroxide in methanol. Heat-induced epitope retrieval was performed for 5 min with 10 mM citrate buffer. Slides were blocked according to the kit manufacturer instructions (N-Histofine® MOUSESTAIN KIT Nichirei Biosciences, Japan) and then incubated overnight at 4 ^o^C with the primary anti-iNOS mAb (clone CXNFT, eBioscience) diluted at 1:100. Sections were then washed, blocked, and treated with the secondary anti-rat IgG F(ab’)^2^ conjugated to polymer-peroxidase (Simple Stain Mouse MAX PO, Nichirei Biosciences) for 1 h. Peroxidase was revealed by using the substrate chromogen system (Dako liquid 3,3’diaminobenzidine (DAB), Dako, Carpinteria, CA, USA). Slides were then counterstained with Harris’ haematoxylin and mounted with coverslips. Images were digitalized with a digital camera (Evolution VF, MediaCybernetics, USA) coupled to a Nikon Eclipse E800 microscope (Nikon Instruments). The images were captured in a blind way by using the interface capture software Q-Capture 2.95.0, version 2.0.5 (Silicon Graphics Inc, USA). To normalize analyses in the mouse groups that show different inflammatory responses in the hearts, 16–24 images/heart were taken from the fields with the presence of inflammatory foci, mainly localized at the pericardium area and at the myocardium near the pericardium (hot spots). For each mouse heart, iNOS^+^ cells were manually counted in the images, whereas the total number of cells was determined by using the ImageJ 1.53k program and expressed as the number of cells/mm^2^.

### Statistics and reproducibility

Data were analysed by using GraphPad Prism (v. 6.0). The results are expressed as the mean and standard error of the mean (SEM) in the figures. The number (n) of animals per group is indicated in the figure or figure legends. Data were first analysed by the Kolmogorov-Smirnov test to assess normal distribution. For parasitemia, data were first transformed to log of parasites per mL for statistical analysis. Two experimental groups or treatments were compared with paired or unpaired Student’s two-tailed *t*-test. One-way analysis of variance (ANOVA) was performed to analyse more than two groups or treatments, followed by Tukey’s or Bonferroni’s post-test in selected pairs of data. Significant differences are indicated for ^*^P < 0.05, ^**^P < 0.01,^***^P < 0.001, and ^****^P < 0.0001. For in vitro experiments, data are expressed as the mean of technical replicates per treatment and SEM. The numbers of independent repeat experiments are indicated in the figure legends. The outliers were removed after Grubbs’ test only in Fig. 6e and Supplementary Fig. 7. The eliminated outliers were shown and highlighted in the Supplementary Data 1 file.

## Supplementary information

Supplementary Information

Description of Additional Supplementary Files

Supplementary Data 1

nr-reporting-summary

## Data Availability

The source data underlying the figures of this manuscript are provided as Supplementary Data 1.
