# Supplementary material for: Axl receptor induces efferocytosis, dampens M1 macrophage responses and promotes heart pathology in Trypanosoma cruzi infection
Source: Commun Biol. 2022 Dec 29;5:1421. doi: 10.1038/s42003-022-04401-w (PMC9800583; doi:10.1038/s42003-022-04401-w)
Supplement: Supplementary file 1 — Supplementary Information [file 42003_2022_4401_MOESM1_ESM.pdf]

Axl receptor induces efferocytosis, dampens M1 macrophage responses and promotes heart pathology in *Trypanosoma cruzi* infection

Thaís S. Rigoni[1], Natália S. Vellozo[1], Kamila Guimarães-Pinto[1,2], Mariela Cabral-Piccin[1], Laryssa Fabiano-Coelho[1], Thayane C. Matos-Silva[1], Alessandra A. Filardy[1,2], Christina M. Takiya[1], Marcela F. Lopes[1*]

[1]Instituto de Biofísica Carlos Chagas Filho, Universidade Federal do Rio de Janeiro, Rio de Janeiro, RJ, Brazil. [2]Instituto de Microbiologia Paulo de Góes, Universidade Federal do Rio de Janeiro, Rio de Janeiro, RJ, Brazil.

***Correspondence:** marcelal@biof.ufrj.br

Supplementary table 1

Supplementary figures:

Supplementary Figure 1

Supplementary Figure 2

Supplementary Figure 3

Supplementary Figure 4

Supplementary Figure 5

Supplementary Figure 6

Supplementary Figure 7

Supplementary Figure 8

Supplementary Figure 9

Supplementary Figure 10

Supplementary Table 1

Antibodies - Flow Cytometry	Manufacturer	Catalog	Lot number	Clone	Conc.
Fc-blocking anti-CD16/CD32	BD Pharmigen	553142	1293770	2.4G2	0.5 mg/mL
Allophycocyanin-labelled anti-CD4	ebioscience	17-0041-83	E004011	GK1.5	0.2 mg/mL
Allophycocyanin-labelled anti-CD8a	ebioscience	17-0081-82	E07056-158	53-6.7	0.2 mg/mL
Allophycocyanin-Cy7 labelled anti-CD8a	BD Pharmigen	557654	1203384	53-6.7	0.2 mg/mL
Allophycocyanin-Cy7 labelled control Rat IgG2a	BD Pharmigen	400523	B233351	RTK2758	0.2 mg/mL
Allophycocyanin-labelled anti-CD19	BD Pharmigen	550992	4944	1D3	0.2 mg/mL
Allophycocyanin-labelled Anti-Axl	R&D	FAB8541A	ADGY0216111	175128	100 tests/mL
Allophycocyanin-labelled anti-CD301 (MGL1/2)	Biolegend	145708	B297344	LOM-14	0.2 mg/mL
Allophycocyanin-labelled control Rat IgG2b	Biolegend	400612	B202123	RTK4530	0.2 mg/mL
Allophycocyanin-labelled anti-TCR β chain	BD Pharmigen	553174	0000075631	H57-597	0.2 mg/mL
Allophycocyanin-labelled anti-CD11b	ebioscience	17-0112-82	4295321	M1/70	0.2 mg/mL
Allophycocyanin-labelled anti-Ly6C	ebioscience	17-5932-82	E10761-1632	HK1.4	0.2 mg/mL
Allophycocyanin-labelled anti-F4/80	Biolegend	123116	B298926	BM8	0.2 mg/mL
PE-CF594-labelled anti-CD11b	BD horizon	562287	2026039	M1/70	0.2 mg/mL
PE-labelled anti-CD11b	BD Pharmigen	553311	68320	M1/70	0.2 mg/mL
PE-labelled anti-IL-12(p35)	R&D	IC2191P	ABAP0419051	27537	100 tests/mL
PE-labelled control murine IgG1	R&D	IC002P		11711	100 tests/mL
PE-labelled anti-NOS2	ebioscience	12-5920-82	E17914-105	CXNFT	0.2 mg/mL
PE-labelled anti-MerTK	ebioscience	12-5751-82	4310607	DS5MMER	0.2 mg/mL
PE-labelled control rat IgG2a	ebioscience	12-4321-82		eBR2a	0.2 mg/mL
FITC-labelled anti-Ly6C	Biolegend	128006	B195146	HK1.4	0.5 mg/mL
FITC-labelled anti-F4/80	ebioscience	11-4801-81	E00610-1314	BM8	0.5 mg/ml
FITC-labelled anti-Arginase 1	R&D	IC5868F	ABPT0215041	polyclonal	100 tests/mL
FITC-labelled control sheep IgG	R&D	IC016F	AAUM02	polyclonal	100 tests/mL
FITC-labelled Annexin V	R&D	4830-01-1			1 µL/test (125
FITC-labelled Annexin V	BD Pharmigen	556419	66134		5 µL/test (200
7-AAD viability staining solution	ebioscience	00-6993-50	2018163		50 µg/mL
CFSE	Invitrogen	C34554	29533W		5 mM
Viability Dye eFluor 450	ebioscience	65-0863-14	4300716		1µL/test (100
**KITs for ELISA and IHC**	**Manufacturer**	**Catalog**	**Lot number**		
anti-IL-12p70	Peprotech	900-T97	1006S97		
anti-TNF-α	Peprotech	900-TM54	121054-1		
anti-IL-10	Peprotech	900-T53	51453		
anti-IFN-γ	Peprotech	900-TM98	0713AFC98		
anti-CXCL9	R&D	DY492			
anti-CCL17	R&D	DY529			
N-Histofine® MOUSESTAIN KIT	Nichirei	414322F			
Polymer peroxidase-anti-rat IgG F(ab')2 N-Histofine® Simple Stain Mouse MAX PO	Nichirei Biosciences	414311F			
rm MER/Fc Chimera recombinat mouse	R&D	591-MR	CBP1216111		100 µg/mL

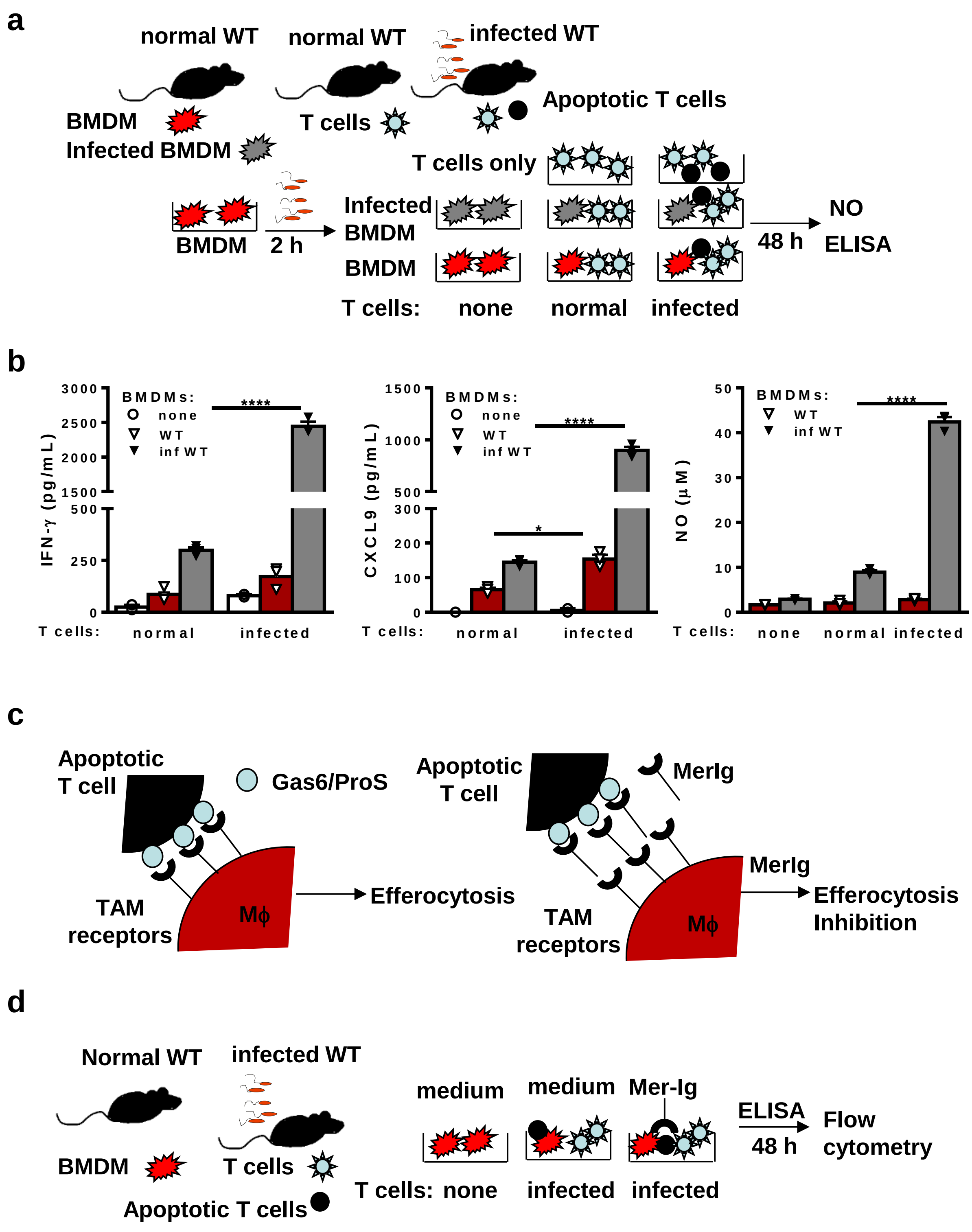
a
normal WT
normal WT
infected WT
BMDM
Infected BMDM
T cells
Apoptotic T cells
T cells only
Infected BMDM
BMDM
BMDM
2 h
48 h
NO
ELISA
T cells: none normal infected
b
BMDMs:
none
WT
inf WT
****
*
IFN-γ (pg/mL)
CXCL9 (pg/mL)
NO (μM)
T cells: normal infected
T cells: none normal infected
c
Apoptotic T cell
Gas6/ProS
TAM receptors
Mϕ
Efferocytosis
MerIg
Efferocytosis Inhibition
d
Normal WT
infected WT
BMDM
T cells
Apoptotic T cells
medium
medium
Mer-Ig
ELISA
48 h
Flow cytometry
T cells: none infected infected

Supplementary Figure 1. Macrophage activation in T-cell cocultures and schematic view of TAM receptor inhibition. **a** BMDMs from B6 (WT) mice were cultured in medium only or infected for 2 h at a 10:1 metacyclic trypomastigote/macrophage ratio, washed and then cultured in triplicate in medium only or with T cells from normal or infected WT mice. After 48 h, the supernatants were analysed for IFN-γ, CXCL9 and NO. **b** Graphs depict IFN-γ and CXCL9 produced in T-cell cultures in the absence (open circles) or presence of infected (closed triangles) or uninfected (open triangles) macrophages. NO was detected in macrophages cultured in the absence (none) or presence of T cells from normal or infected mice. The results represent at least 3 independent experiments and are expressed as the means and SEM of n=3 technical replicates. The results were analysed by one-way ANOVA followed by Bonferroni posttest of selected pairs of data (macrophages cultured with T cells from normal *versus* infected mice). Significant differences are indicated as (*) for $P<0.05$ and (****) $P<0.0001$. **c** The TAM receptor inhibitor Mer-Ig binds to the bridge proteins Gas6 and ProS on the surface of apoptotic cells and prevents efferocytosis through the TAM receptors Tyro3, Axl, and Mer. **d** BMDMs from WT mice were cultured in medium only or with T cells from infected mice in the presence or absence of Mer-Ig for 48 h, washed, and then analysed by flow cytometry. Supernatants were removed for ELISA assays.

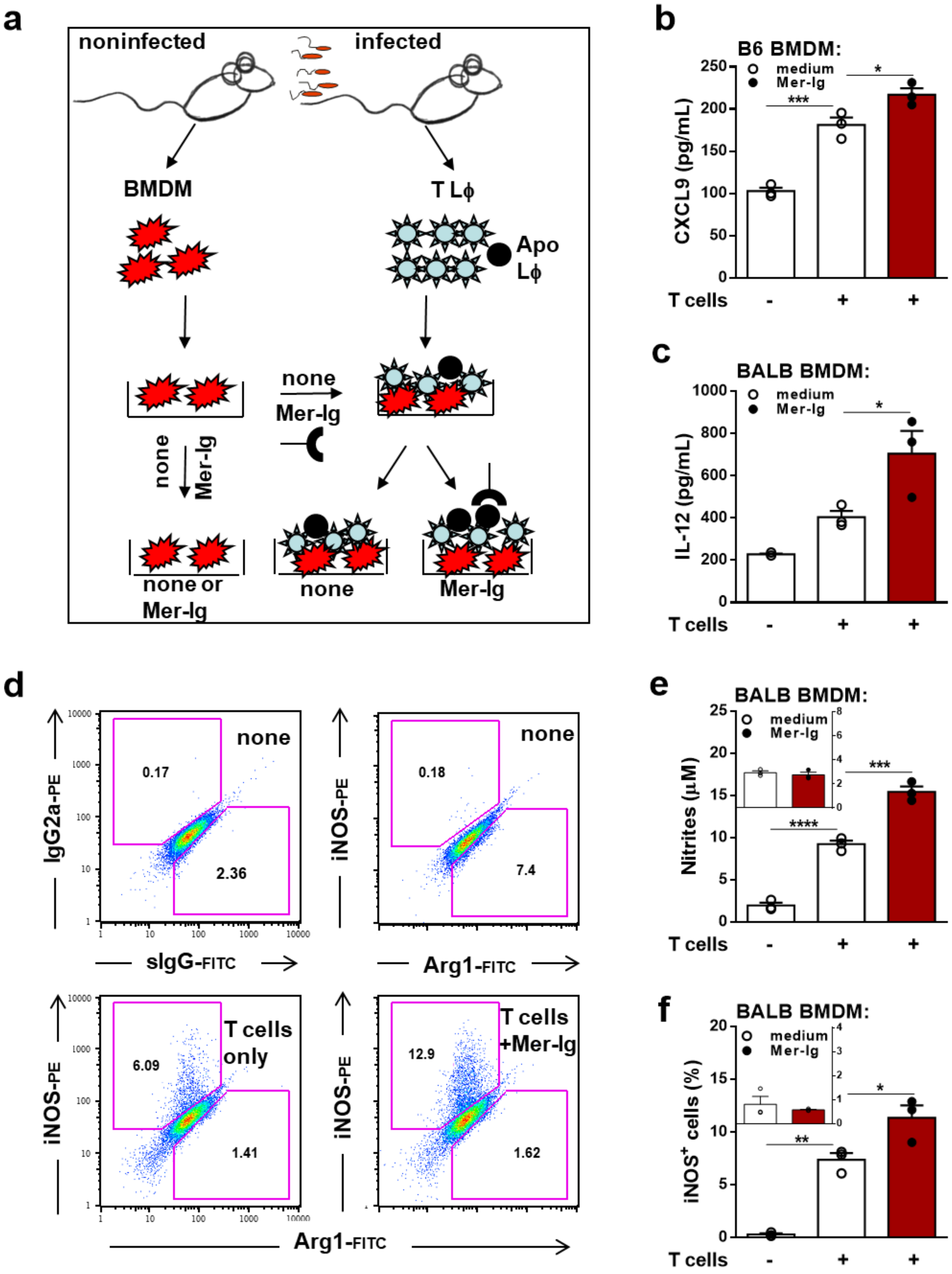
a
noninfected
infected
BMDM
T Lϕ
Apo
Lϕ
none
Mer-Ig
none
Mer-Ig
none or
Mer-Ig
none
Mer-Ig
b
B6 BMDM:
medium
Mer-Ig
CXCL9 (pg/mL)
T cells
c
BALB BMDM:
medium
Mer-Ig
IL-12 (pg/mL)
T cells
d
IgG2a-PE
sIgG-FITC
none
0.17
2.36
iNOS-PE
Arg1-FITC
none
0.18
7.4
iNOS-PE
T cells
only
6.09
1.41
iNOS-PE
T cells
+Mer-Ig
12.9
1.62
Arg1-FITC
e
BALB BMDM:
medium
Mer-Ig
Nitrites (μM)
T cells
f
BALB BMDM:
medium
Mer-Ig
iNOS+ cells (%)
T cells

Supplementary Figure 2. The blockade of TAM receptors improves M1 responses to T cells from Th2-prone BALB/c or Th1-prone B6 mice. **a** BMDMs from B6 (**b**) or BALB/c mice were treated (closed circles) or not (open circles) with Mer-Ig and then cocultured in triplicate with T cells from *T. cruzi*-infected (18 dpi) mice (from the same strain) for 48 h. Supernatants were collected for **b**, **c** ELISA and **e** NO assays. **d**, **f** Macrophages from BALB/c mice were washed to remove T cells and then stained for flow cytometry. Gated F4/80$^+$ cells were then analysed for intracellular iNOS and Arg1 expression or control isotypes. The results of one experiment with BALB/c mice are expressed as the means and SEM of n=3 technical replicates and corroborate B6 results (Fig. 1 represents 2 repeat experiments with B6 mice). The results were analysed by ANOVA followed by Bonferroni posttest of selected pairs of data (macrophages cultured alone *versus* those cocultured with T cells; macrophages cocultured with T cells in the absence of treatment *versus* those treated with Mer-Ig). **Insets:** Data analysed by unpaired Student's *t*-test comparing macrophages cultured in medium only or treated with Mer-Ig. Significant differences are indicated as (*) for P<0.05, (**) P<0.01, (***) P<0.001, and (****) P<0.0001.

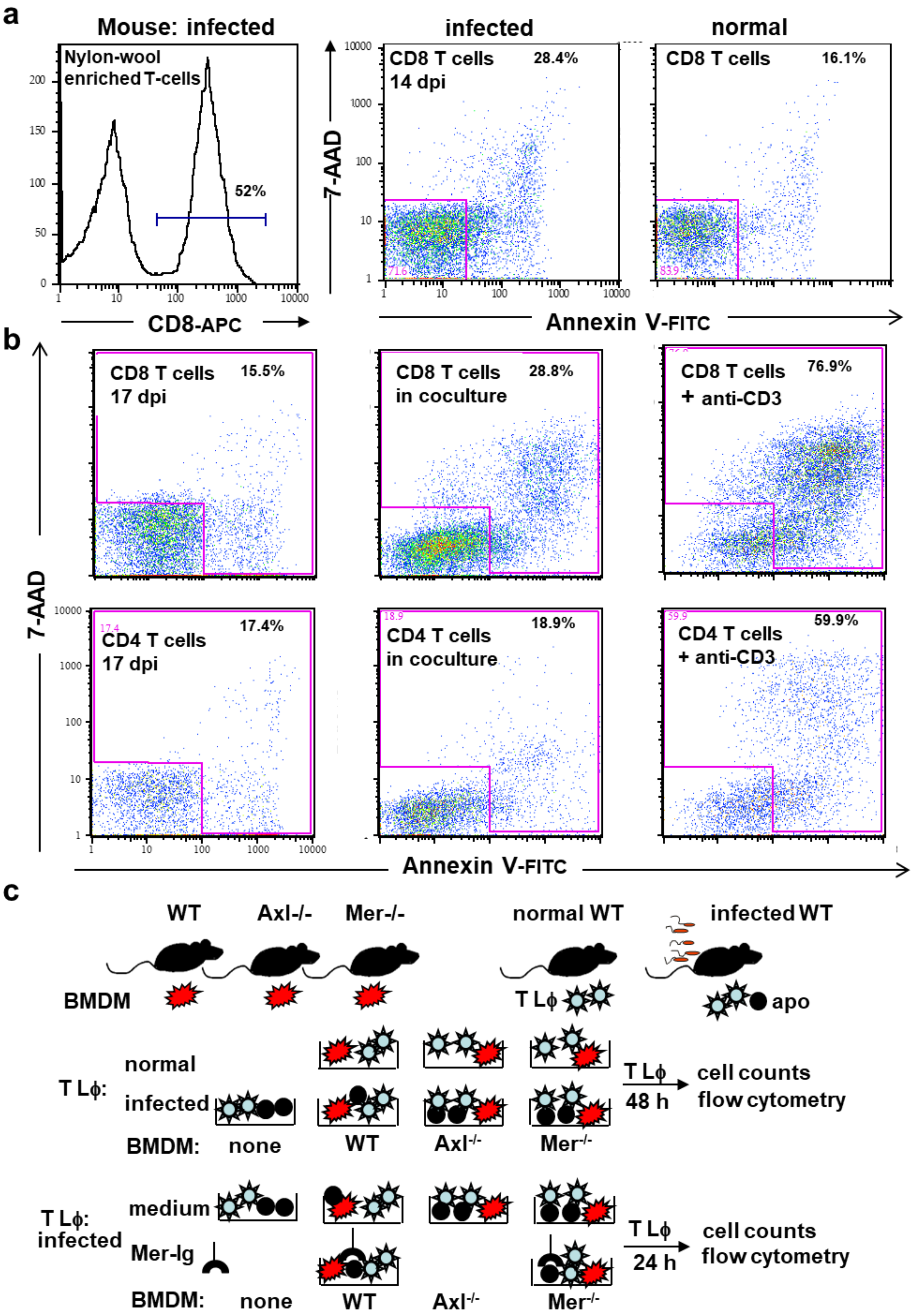
a
Mouse: infected
Nylon-wool enriched T-cells
52%
CD8-APC
infected
normal
CD8 T cells 14 dpi
28.4%
CD8 T cells
16.1%
7-AAD
Annexin V-FITC
b
CD8 T cells 17 dpi
15.5%
CD8 T cells in coculture
28.8%
CD8 T cells + anti-CD3
76.9%
CD4 T cells 17 dpi
17.4%
CD4 T cells in coculture
18.9%
CD4 T cells + anti-CD3
59.9%
7-AAD
Annexin V-FITC
c
WT
Axl-/-
Mer-/-
normal WT
infected WT
BMDM
T Lϕ
apo
T Lϕ:
normal
infected
BMDM: none WT Axl-/- Mer-/-
T Lϕ 48 h
cell counts flow cytometry
T Lϕ: infected
medium
Mer-Ig
BMDM: none WT Axl-/- Mer-/-
T Lϕ 24 h
cell counts flow cytometry

Supplementary Figure 3. Proapoptotic T cells from *T. cruzi*-infected mice in efferocytosis assays. Splenocytes from normal or ip-infected WT mice at **a** 14 or **b** 17 dpi were first enriched in nylon wool columns and then stained with anti-CD4, anti-CD8, AnV, and 7-AAD either **a, b** prior to or **b** after 48 h of culture in the presence of WT BMDMs. Some cocultures were stimulated with soluble anti-CD3 as a positive control for apoptosis induction. The results represent at least 3 independent experiments**.** **c** Schematic view of efferocytosis assays. BMDMs from WT, $Axl^{-/-}$ and $Mer^{-/-}$ mice were cocultured with T cells from normal or infected WT mice. After 48 h, T cells were collected, counted, and stained for apoptosis assessment by flow cytometry. Alternatively, BMDMs were cocultured with T cells from infected mice in the presence or absence of the TAM inhibitor Mer-Ig for 24 h before analyses.

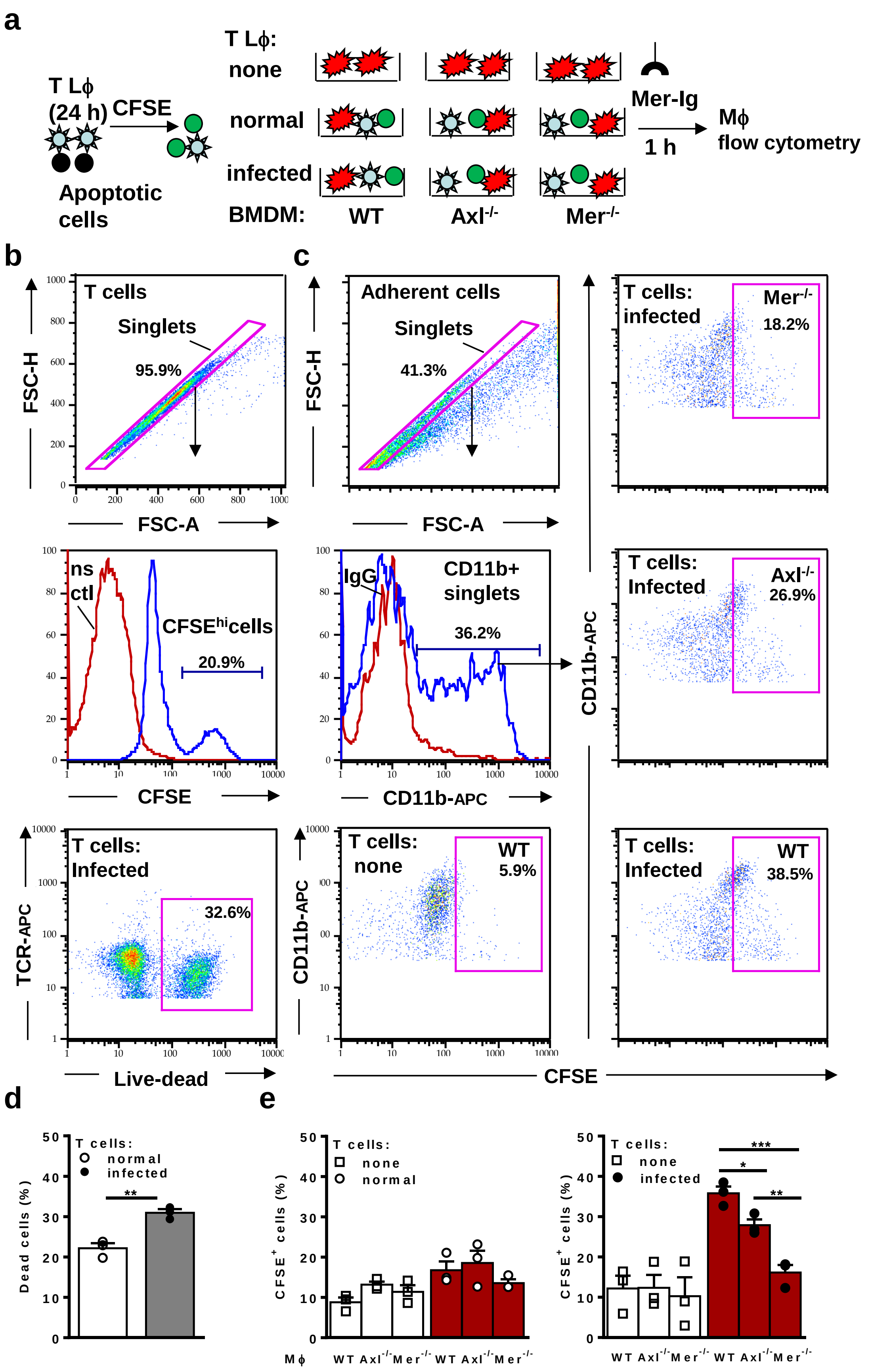
a
T Lϕ (24 h)
CFSE
Apoptotic cells
T Lϕ:
none
normal
infected
BMDM:
WT
Axl-/-
Mer-/-
Mer-Ig
1 h
Mϕ flow cytometry
b
T cells
Singlets
95.9%
FSC-H
FSC-A
ns ctl
CFSEhicells
20.9%
CFSE
T cells: Infected
32.6%
TCR-APC
Live-dead
c
Adherent cells
Singlets
41.3%
IgG
CD11b+ singlets
36.2%
CD11b-APC
T cells: none
WT
5.9%
T cells: infected
Mer-/-
18.2%
T cells: Infected
Axl-/-
26.9%
WT
38.5%
CFSE
d
T cells:
normal
infected
Dead cells (%)
e
T cells:
none
normal
infected
CFSE+ cells (%)
Mϕ
WT Axl-/- Mer-/- WT Axl-/- Mer-/-

Supplementary Figure 4. Defective efferocytosis by Axl$^{-/-}$ and Mer$^{-/-}$ macrophages. **a** Experimental design: BMDMs from WT, Axl$^{-/-}$ and Mer$^{-/-}$ mice were cocultured in triplicate in medium alone (open squares) or with CFSE-labelled T cells from naïve (open circles) or *T. cruzi*-infected (closed circles) WT mice. **b, d** T cells from normal or infected (15 dpi) WT mice were first cultured for 24 h, labelled with CFSE, and then stained with anti-CD4, anti-CD8, anti-TCRβ, and live/dead reagent. **d** Percentages of dead TCRβ^{+} cells in T-cell cultures from normal and infected mice (apoptosis was ascertained by AnV^{+}7-AAD^{+} staining). **c, e** BMDMs were treated for 1 h with CFSE-labelled cells. The supernatants and nonadherent cells were then removed, and adherent macrophages were collected, stained, and gated for singlets and CD11b^{+} cells. CD11b^{+} macrophages were then analysed for CFSE^{+} staining to reveal intracellular T cells. The selection of CFSE^{+} cells was based on the negative control of macrophages treated with medium only. **e** Percentages of macrophages bearing intracellular CFSE^{+} cells. The results represent 2 independent experiments and are expressed as the means and SEM of n=3 technical replicates. Significant differences, as analysed by **d** unpaired Student's *t*-tests and by **e** ANOVA with Tukey's posttest, are indicated as (*) for $P<0.05$, (**) $P<0.01$, and (***) $P<0.001$.

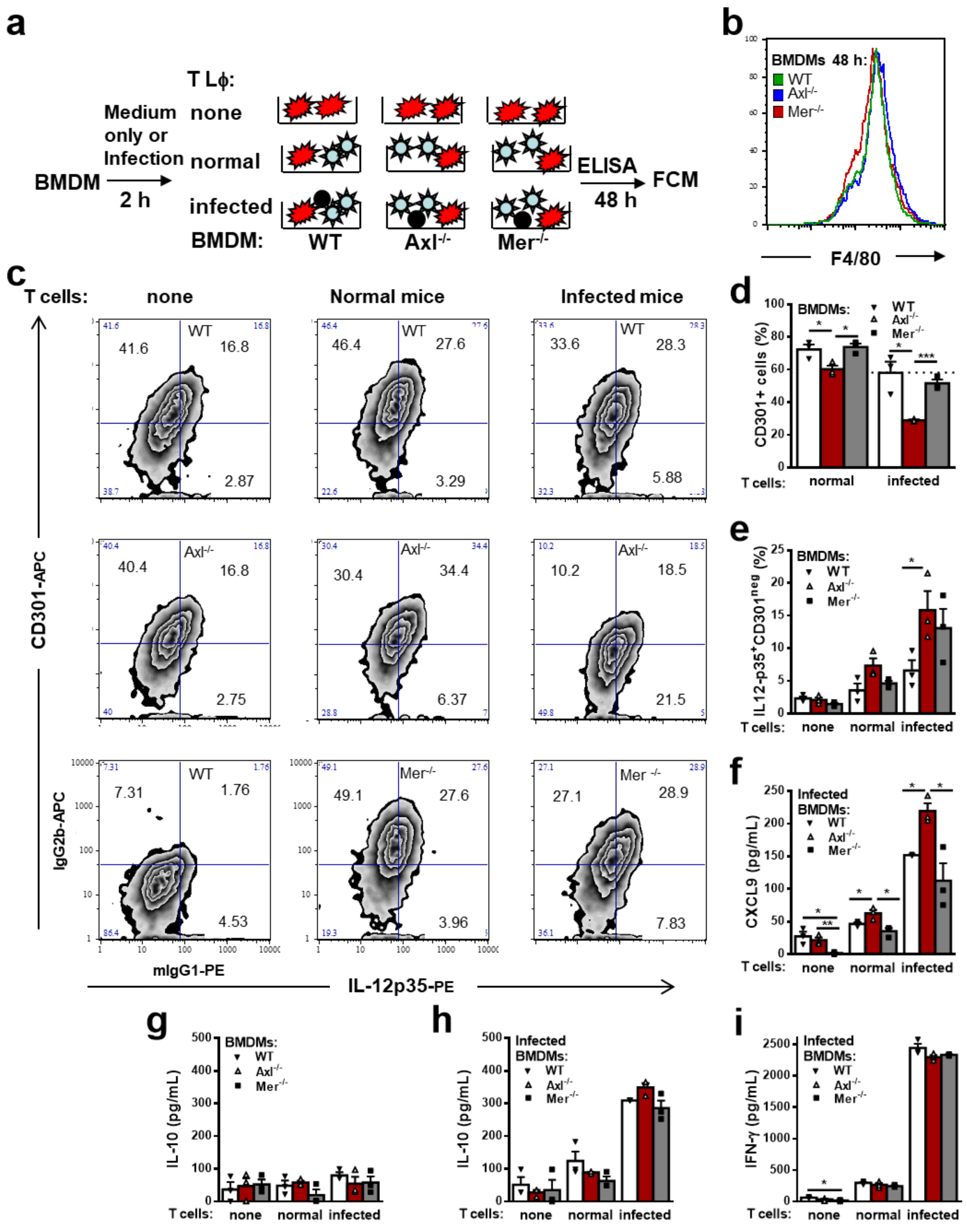

a
T Lϕ:
Medium only or Infection
none
normal
infected
BMDM
2 h
BMDM:
WT
Axl-/-
Mer-/-
ELISA
FCM
48 h
b
BMDMs 48 h:
WT
Axl-/-
Mer-/-
F4/80
c
T cells:
none
Normal mice
Infected mice
CD301-APC
IgG2b-APC
mIgG1-PE
IL-12p35-PE
d
BMDMs:
CD301+ cells (%)
T cells:
normal
infected
e
IL12-p35+CD301neg (%)
none
f
Infected BMDMs:
CXCL9 (pg/mL)
g
IL-10 (pg/mL)
h
i
IFN-γ (pg/mL)

Supplementary Figure 5. Phenotyping of Axl- and Mer-defective macrophages in coculture with T cells. **a** BMDMs from WT (closed triangles), $Axl^{-/-}$ (open triangles) and $Mer^{-/-}$ (closed squares) mice were cultured in triplicate in medium only or infected for 2 h, washed, and then treated with T cells from normal or infected WT mice for 48 h before flow cytometry (FCM) and cytokine analyses. **b** BMDMs from WT (green), $Axl^{-/-}$ (blue) and $Mer^{-/-}$ (red) were analysed for F4/80 expression after 48 h of culture in medium only. **c** Macrophages were gated for F4/80 and then analysed for surface CD301 and intracellular IL-12p35 expression, based on the exclusion of background staining with immunoglobulin G (IgG) isotype controls. **d, e** Graphs depict CD301 and IL-12p35 ($CD301^{neg}$) expression; the dotted line represents CD301 expression in BMDMs cultured with medium only. **f**, **g, h, i** Macrophages were either **g** cultured with T cells or first infected (**f, h, i**) and then cultured with T cells. After 48 h, the culture supernatants were evaluated for the chemokine CXCL9, IL-10 and IFN-γ by ELISA. The results are expressed as the means and SEM of n=3 technical replicates. Significant differences are indicated for $P<0.05$ (*), $P<0.01$ (**), and $P<0.001$ (***), as analysed by unpaired Student's *t*-test between different macrophages. The results are representative of 2 independent experiments.

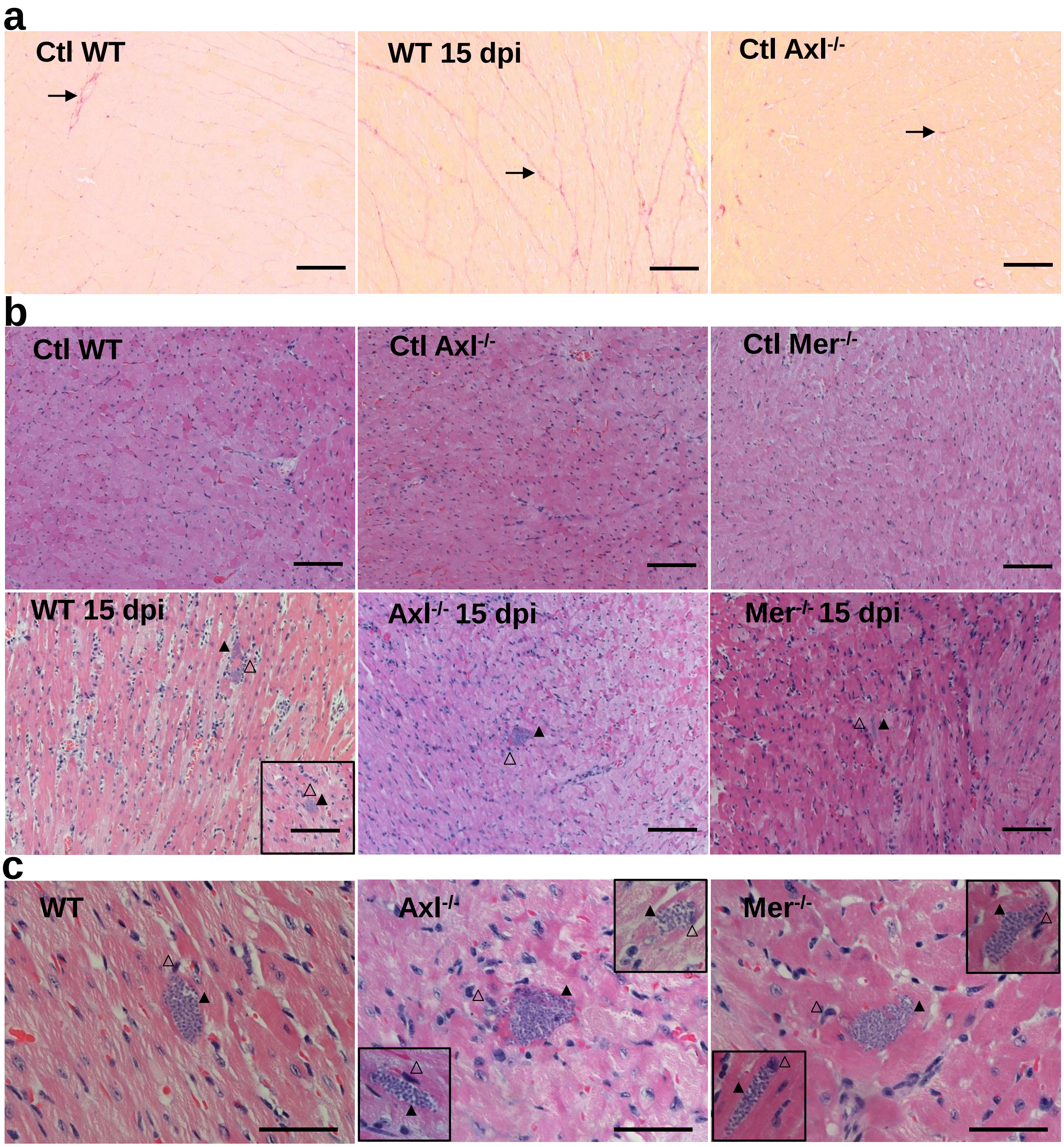
a
Ctl WT
WT 15 dpi
Ctl Axl-/-
b
Ctl WT
Ctl Axl-/-
Ctl Mer-/-
WT 15 dpi
Axl-/- 15 dpi
Mer-/- 15 dpi
c
WT
Axl-/-
Mer-/-

Supplementary Figure 6. Reduced heart pathology in infected Axl-defective mice. a Representative micrographs of **a** picrosirius red- and **b**, **c** H&E-stained heart sections from normal and infected WT, $Axl^{-/-}$ and $Mer^{-/-}$ mice (15 dpi). Bars: 100 µM (**a** and **b**) or 50 µM (**c**). **a** Arrows point to areas with fibrosis; **b**, **c** open arrowheads point to inflammatory cells in close proximity to parasite nests marked with closed arrowheads. **Insets:** Micrograph details show parasite nests and inflammatory cells **b** from the same heart section or **c** from different mice.

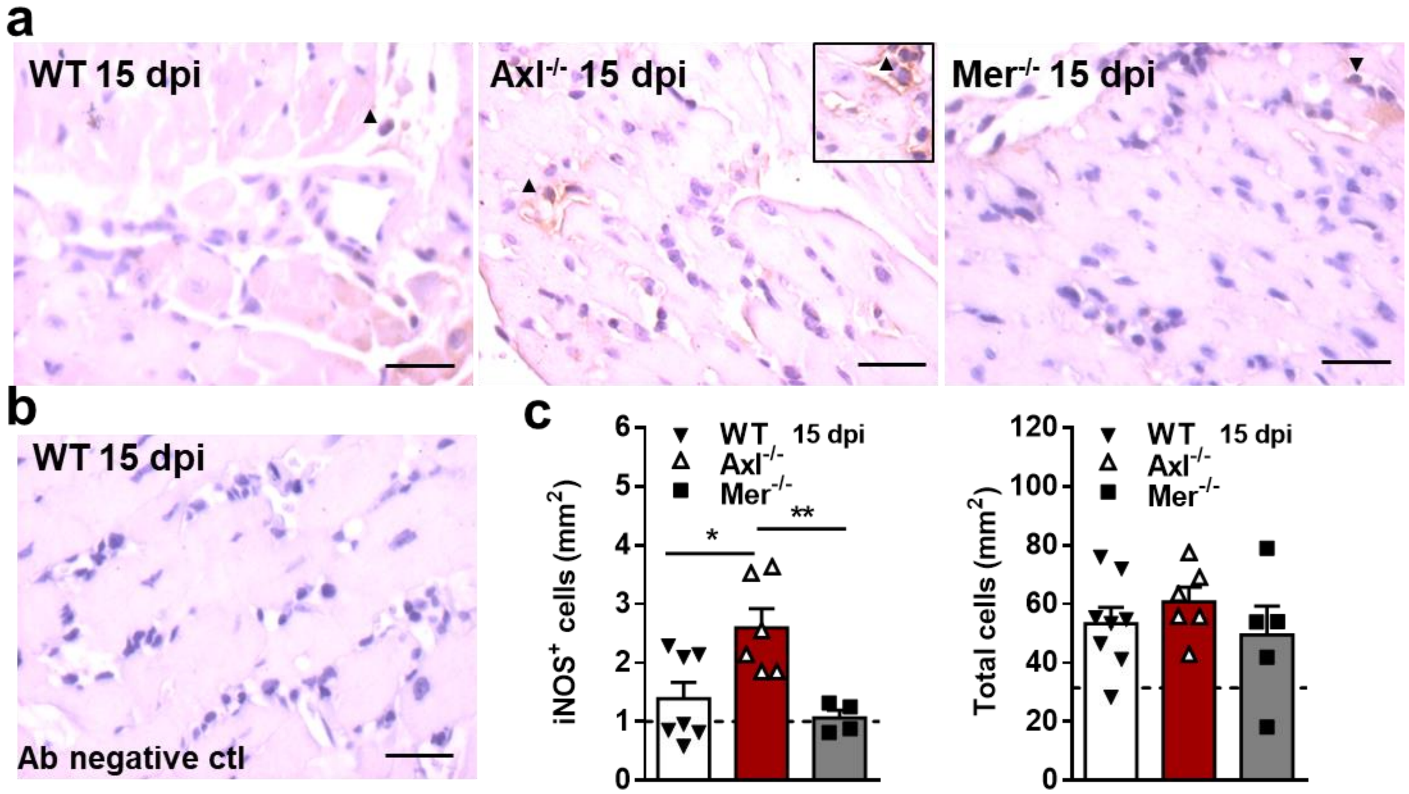

Supplementary Figure 7. Improved immune response in infected Axl-defective mice. a, b Representative micrographs of heart sections from 15 dpi-infected WT, Axl$^{-/-}$ and Mer$^{-/-}$ mice show **a** iNOS^{+} inflammatory cells and **b** a reaction negative control from a infected WT mouse (without primary-antibody). Bar: 50 µM. **a** Arrow heads point to iNOS^{+} cells. **c** Graphs represent iNOS^{+} cells and total number of cells within inflammatory infiltrates in the hearts from WT (closed triangles), Axl$^{-/-}$ (open triangles) and Mer$^{-/-}$ (closed squares). Dashed lines represent basal levels in naïve Axl$^{-/-}$ mice. The results are expressed as the means and SEM for each experimental group; WT (n=8), Axl$^{-/-}$ (n=6), and Mer$^{-/-}$ (n=5) infected mice. Significant differences in unpaired Student's *t*-tests are indicated for P<0.05 (*) and P<0.01 (**). The outliers in iNOS analyses were removed from the infected WT and Mer$^{-/-}$ groups after Grubbs' test.

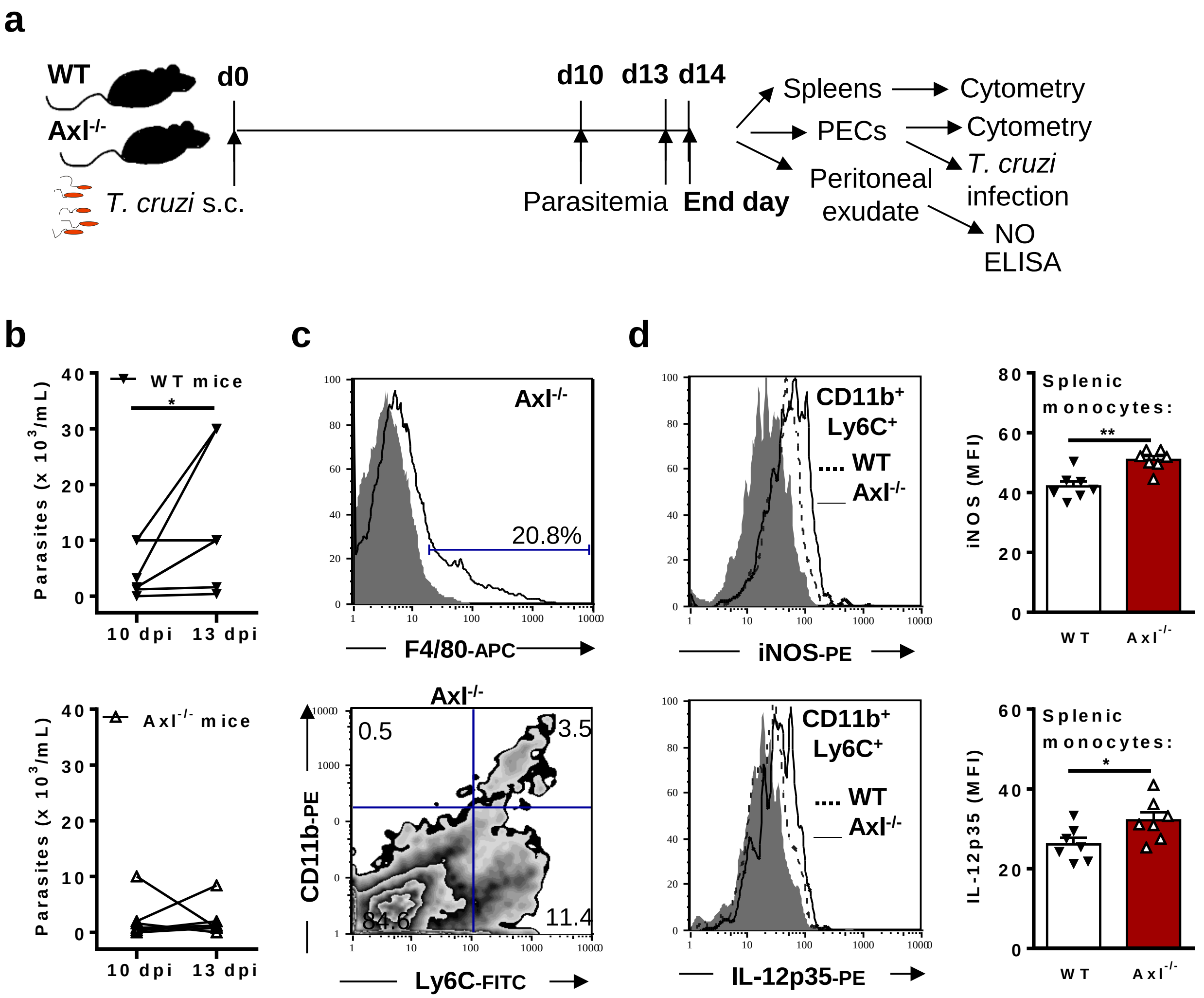

Supplementary Figure 8. Experimental design and effector phenotypes of monocytes. a Male WT (closed triangles) and Axl$^{-/-}$ (open triangles) mice were infected sc with *T. cruzi* parasites. **b** WT and Axl$^{-/-}$ mice were analysed for parasitemia on the indicated days. **c** Flow cytometry figures show gates for F4/80^{+} splenocytes and CD11b^{+}Ly6C^{+} monocytes. **d** Splenocytes were first stained with APC-anti-CD11b and FITC-labelled anti-Ly6C and then processed for intracellular staining with PE-labelled anti-iNOS or anti-IL-12p35. Histograms represent iNOS and IL-12p35 expression in CD11b^{+}Ly6C^{+} splenocytes from WT (dashed lines) and Axl$^{-/-}$ (closed lines), as well as control isotypes (grey). Graphs depict the means and SEM of n=7 mice/group and represent the MFI (mean fluorescence intensity) for IL-12p35- and iNOS-expressing monocytes. Significant differences, as analysed by **a** paired (10 *versus*

13 dpi) and **d** unpaired (infected WT *versus* infected $Axl^{-/-}$ mice) Student's *t*-tests, are indicated as (*) for P<0.05 and (**) P<0.01.

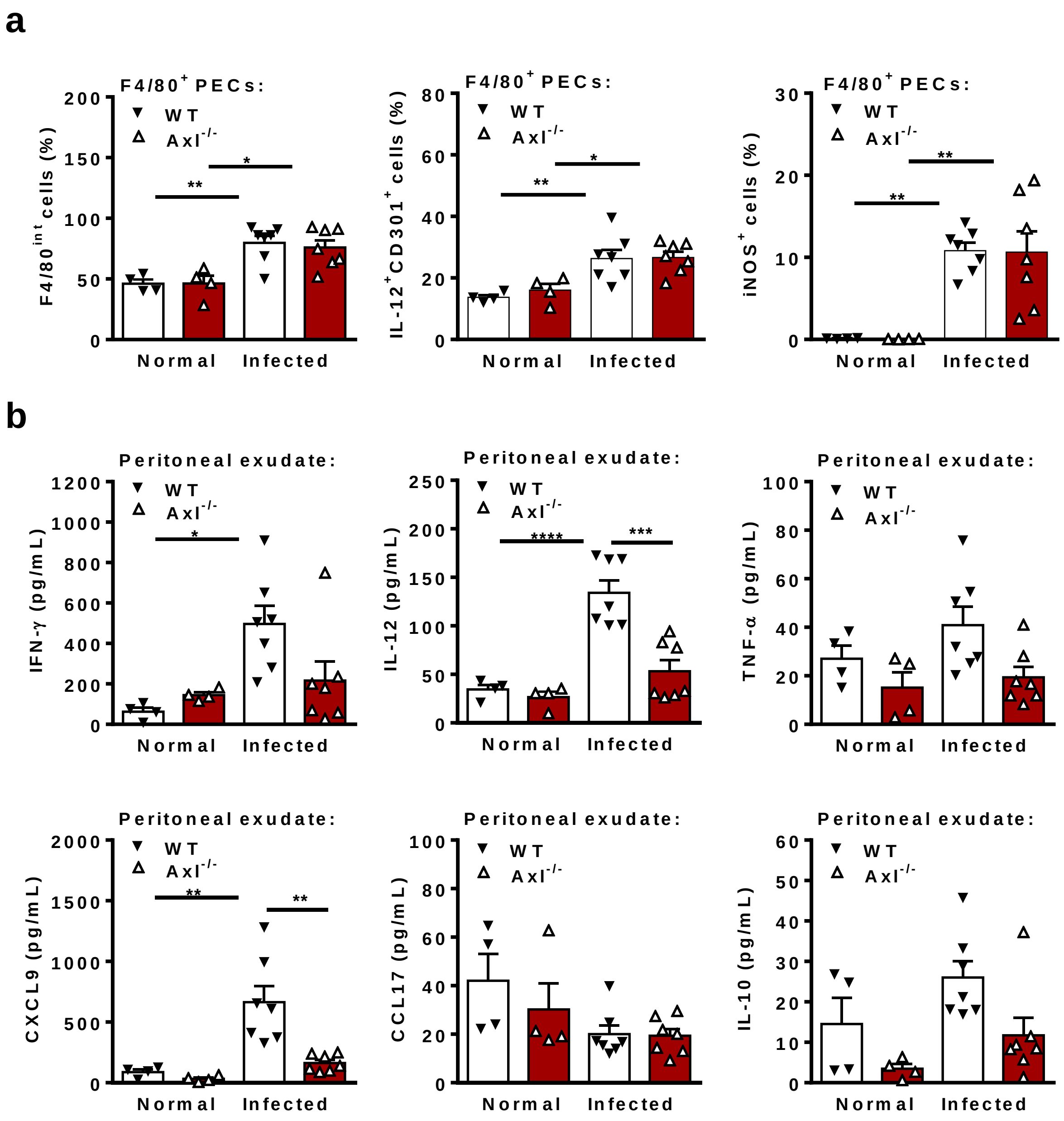

Supplementary Figure 9. Defective Axl engagement does not upregulate systemic immune responses. Male WT (closed triangles) and Axl$^{-/-}$ (open triangles) were infected sc with *T. cruzi* parasites. Normal Axl$^{-/-}$ and WT (n=4) mice were used as controls. **a** The graphs depict the percentages of F4/80^{int} peritoneal macrophages, as well as CD301^{+}IL12p35^{+} macrophages and iNOS^{+} macrophages. **b** Cytokine responses in infected WT and Axl$^{-/-}$ mice, as evaluated in peritoneal exudates obtained by washing with 5 ml of culture medium. The means and SEM of n=4 normal mice and n=7

infected mice/group are presented for each experimental group. Significant differences, as analysed by ANOVA followed by Bonferroni's posttest (noninfected *versus* infected mice; $Axl^{-/-}$ *versus* WT mice), are indicated as (*) for $P<0.05$, (**) $P<0.01$, (***) $P<0.001$, and (****) $P<0.0001$.

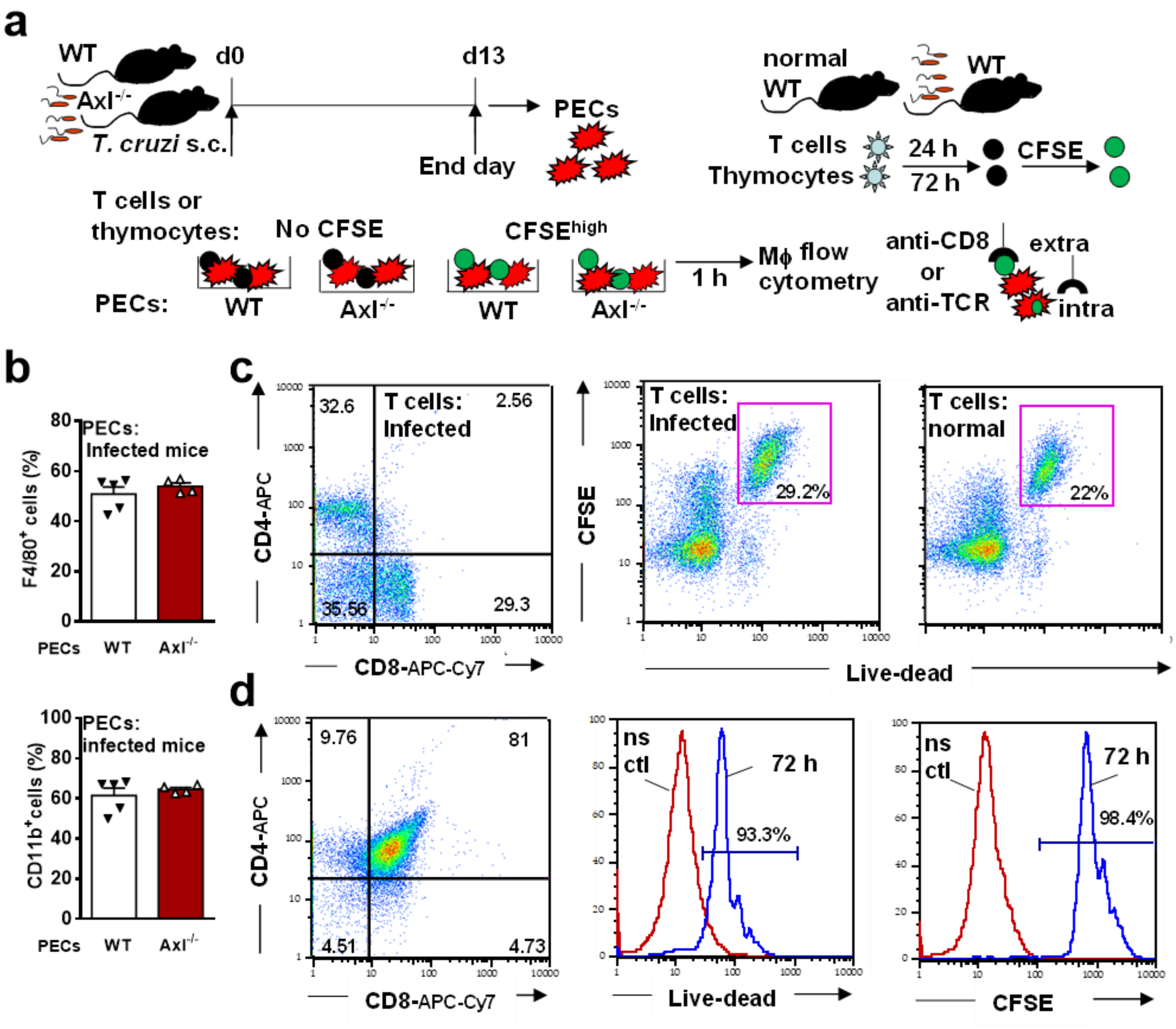

Supplementary Figure 10. Experimental design and preparation of CFSE-labelled cells for efferocytosis assays. a PECs were obtained at 13 dpi from WT (closed triangles) and Axl$^{-/-}$ (open triangles) mice infected sc with *T. cruzi*, whereas CFSE-labelled cells were generated by using thymocytes and naïve T cells from normal mice, and T cells from infected WT mice. PECs were cultured for 24 h and then treated for 1 h with CFSE-negative or CFSE-labelled cells. Anti-TCR or anti-CD8 labelling was employed either to exclude or identify macrophages bound to extracellular cells. **b** PECs taken from infected WT and Axl mice have similar proportions of macrophages stained with FITC anti-F4/80 and APC-CD11b mAbs. Results are expressed as the means and SEM of n=5 WT mice and n=4 Axl$^{-/-}$ mice and analysed by unpaired Student's *t*-tests. **c** Splenic T cells from normal

or infected (13 dpi) WT mice were first cultured for 24 h, labelled with CFSE and then stained with anti-CD4, anti-CD8, and live/dead reagent. Only dead cells from naïve and infected mice yielded CFSE-labelled cells. **d** Thymocytes from naïve mice were first cultured for 72 h, labelled with CFSE, and then stained as above. Thymocytes (most $CD4^+CD8^+$ cells) yielded a homogenous population of dead and highly labelled $CFSE^+$ cells. CFSE-negative thymocytes (not stained control, ns ctl) were used as a negative control for CFSE staining in macrophage flow cytometry assays.
