# Supplementary material for: Axl receptor induces efferocytosis, dampens M1 macrophage responses and promotes heart pathology in Trypanosoma cruzi infection
Source: Commun Biol. 2022 Dec 29;5:1421. doi: 10.1038/s42003-022-04401-w (PMC9800583; doi:10.1038/s42003-022-04401-w)
Supplement: Supplementary file 4 — nr-reporting-summary [file 42003_2022_4401_MOESM4_ESM.pdf]

nature portfolio

Corresponding author(s): Marcela F. Lopes

Last updated by author(s): Dec 11, 2022

Reporting Summary

Nature Portfolio wishes to improve the reproducibility of the work that we publish. This form provides structure for consistency and transparency in reporting. For further information on Nature Portfolio policies, see our Editorial Policies and the Editorial Policy Checklist.

Statistics

For all statistical analyses, confirm that the following items are present in the figure legend, table legend, main text, or Methods section.

n/a	Confirmed	
☐	☒	The exact sample size (*n*) for each experimental group/condition, given as a discrete number and unit of measurement
☐	☒	A statement on whether measurements were taken from distinct samples or whether the same sample was measured repeatedly
☐	☒	The statistical test(s) used AND whether they are one- or two-sided<br>*Only common tests should be described solely by name; describe more complex techniques in the Methods section.*
☐	☒	A description of all covariates tested
☐	☒	A description of any assumptions or corrections, such as tests of normality and adjustment for multiple comparisons
☐	☒	A full description of the statistical parameters including central tendency (e.g. means) or other basic estimates (e.g. regression coefficient) AND variation (e.g. standard deviation) or associated estimates of uncertainty (e.g. confidence intervals)
☐	☒	For null hypothesis testing, the test statistic (e.g. *F*, *t*, *r*) with confidence intervals, effect sizes, degrees of freedom and *P* value noted<br>*Give P values as exact values whenever suitable.*
☒	☐	For Bayesian analysis, information on the choice of priors and Markov chain Monte Carlo settings
☒	☐	For hierarchical and complex designs, identification of the appropriate level for tests and full reporting of outcomes
☒	☐	Estimates of effect sizes (e.g. Cohen's *d*, Pearson's *r*), indicating how they were calculated

Our web collection on statistics for biologists contains articles on many of the points above.

Software and code

Policy information about availability of computer code

Data collection	For flow cytometry, Cell Quest Program (FACS Calibur BD system) was used for data collection. BD FACSDiva (BD LSRFortessa system) was used in the efferocytosis experiments. For generating images, Zeiss Axi Imager D2 software and the interface capture software Q-Capture 2.95.0, version 2.0.5 (Silicon Graphics Inc, USA) were used; for plate spectrophotometer, Molecular Device Spectramax M5 software was used.
Data analysis	TreeStar FlowJow software (version 3.7) was used for flow cytometry data analyses; NIH ImageJ (1.53K) program for histopathology and IHC analyses; GraphPad Prism (v. 6.0) for statistics.

For manuscripts utilizing custom algorithms or software that are central to the research but not yet described in published literature, software must be made available to editors and reviewers. We strongly encourage code deposition in a community repository (e.g. GitHub). See the Nature Portfolio guidelines for submitting code & software for further information.

Data

Policy information about availability of data

All manuscripts must include a data availability statement. This statement should provide the following information, where applicable:

- Accession codes, unique identifiers, or web links for publicly available datasets
- A description of any restrictions on data availability
- For clinical datasets or third party data, please ensure that the statement adheres to our policy

The source data underlying the figures of this manuscript are provided as Supplementary Data 1.

Human research participants

Policy information about studies involving human research participants and Sex and Gender in Research.

Reporting on sex and gender	the study did not invove human research.
Population characteristics	n/a
Recruitment	n/a
Ethics oversight	n/a

Note that full information on the approval of the study protocol must also be provided in the manuscript.

Field-specific reporting

Please select the one below that is the best fit for your research. If you are not sure, read the appropriate sections before making your selection.

☒ Life sciences ☐ Behavioural & social sciences ☐ Ecological, evolutionary & environmental sciences

For a reference copy of the document with all sections, see nature.com/documents/nr-reporting-summary-flat.pdf

Life sciences study design

All studies must disclose on these points even when the disclosure is negative.

Sample size	No calculation was used to predefine sample size. For in vivo studies, mouse groups of 4-8 were used according to availability of mouse genotypes in the colony, further restricted during COVID-19 pandemics. Because multiple groups were included (infected, non infected, different genotypes) and multiple samples and manipulations (spleen, PECs, cell culture, flow cytometry), a handling of about 20 mouse/ experiment is at our maximum experimental capacity. An n=5 also allowed tests for normal distribution for each variable
Data exclusions	All data sets were included, even outliers in analyses, and no exclusion criteria were pre-established other than samples lost for any technical reason. An exception is an outlier identified in the infected WT group in Figure 6E and 2 outliers (WT and Mer-/- groups) in Supplementary Fig. 7, which were then removed after Grubbs's test. A statement was included in the method section and in each legend; the removed results were included and highlighted in the Supplementary Data 1 file.
Replication	The numbers of repeat experiment and the n of mice/experimental group are included in the legends. Experiments in vitro were repeated at least twice, in average 3 times, except the single BALB/c experiment in supplementary figure 1, which corroborates the findings with B6 mice experiments depicted in Figure 1. Of course, some intents of replication did not work for technical reasons throughout the study.
Randomization	Groups were matched by age, sex, and genetic background. Mice were distributed randomically in the control and infected groups before infection. Then they were identified for individual determination of parasitemia and each experimental variable. No animal was excluded from sampling and analyses.
Blinding	The collection of data was not done in a predicted blinding fashion, except for the blind acquisition of photographs. However, several authors perfomed different parts of the experiment guided only by the sample numbers and not necessarily aware of the correspondence between number and mouse group during the experiment.

Reporting for specific materials, systems and methods

We require information from authors about some types of materials, experimental systems and methods used in many studies. Here, indicate whether each material, system or method listed is relevant to your study. If you are not sure if a list item applies to your research, read the appropriate section before selecting a response.

Materials & experimental systems

n/a	Involved in the study	
☐	☒	Antibodies
☒	☐	Eukaryotic cell lines
☒	☐	Palaeontology and archaeology
☐	☒	Animals and other organisms
☒	☐	Clinical data
☒	☐	Dual use research of concern

Methods

n/a	Involved in the study	
☒	☐	ChIP-seq
☐	☒	Flow cytometry
☒	☐	MRI-based neuroimaging

Antibodies

Antibodies used	We provided the Supplementary table 1 in the Supplementary information file which includes detailed information about the antibodies and reagents used in flow cytometry, ELISA, and IHC studies.
Validation	All antibodies used are comercially available and for use in mouse experimentation for flow cytometry, IHC or ELISA, according to datasheet information.

Animals and other research organisms

Policy information about studies involving animals; ARRIVE guidelines recommended for reporting animal research, and Sex and Gender in Research

Laboratory animals	Mus musculus (mouse) BALB/c and C57BL/6 (B6) mice; Axl-/- (strain #011121) and Mertk-/- (strain #011122) mice (1) deposited at the Jackson Laboratory, which were backcrossed to B6 background for at least 6 and 9 generations, respectively, according to the original groups (Lu, Q. & Lemke, G. Homeostatic regulation of the immune system by receptor tyrosine kinases of the Tyro 3 family. Science 293, 306-311, 2001).
Wild animals	The study did not involve wild animals.
Reporting on sex	For infection studies, male mice were used because the background B6 is relatively resistant to infection and females are more resistant than males to Trypanosoma cruzi infection. For the use of naive mice or BMDMs, both female and male mice were included because gender does not influence results.
Field-collected samples	The study did not involve samples collected from the field.
Ethics oversight	The animal study was reviewed and approved by the Ethics Committee for Use of Animals at the Federal University of Rio de Janeiro (CEUA-UFRJ). All experiments were conducted as in the protocols 078/16 and A22/19-078-16, according to national and institutional regulations that comprise with international standards.

Note that full information on the approval of the study protocol must also be provided in the manuscript.

Flow Cytometry

Plots

Confirm that:

☒ The axis labels state the marker and fluorochrome used (e.g. CD4-FITC).

☒ The axis scales are clearly visible. Include numbers along axes only for bottom left plot of group (a 'group' is an analysis of identical markers).

☒ All plots are contour plots with outliers or pseudocolor plots.

☒ A numerical value for number of cells or percentage (with statistics) is provided.

Methodology

Sample preparation	Freshly-isolated murine cells (PECs, splenocytes), Nylon-wool enriched T cells, cultured cells (BMDMs, T cells) were counted, washed with FACS buffer, treated with normal mouse serum or anti-CD16/CD32 for Fc blocking and then stained with labeled antibodies. For intracellular staining, cells were permeabilized and fixed before staining. For annexin V staining, a specific buffer was used, according to manufacturer and 7-AAD was added just prior to flow cytometry.
Instrument	FACS Calibur BD system and BD LSRFortessa system.
Software	BD Cell Quest Program and BD FACSDiva for collection of data; TreeStar FlowJow software (version 3.7) for data analyses.
Cell population abundance	We did not perform any cell sorting. Nylon-wool enriched T-cells are overall 75-85% CD4+ and CD8+ T cells, which were analyzed within CD4+ and CD8+ cell gates.
Gating strategy	For most experiments, initial SSC/FSC gates included all cell populations; then, specific cell populations stained by labelled antibodies were defined in histograms or dot plots, by using unstained cells or control isotype staining to define negative populations. In the next step, gated cell populations were analysed for specific properties with labelled antibodies based on the exclusion of negative cells, by using labelled isotype control antibodies to design gates. Gate strategies were included in the figures where they were relevant. In efferocytosis experiments, SSC/FSC gate strategy was followed by exclusion of doublets in a FSC-A/FSC-H gate before gating macrophages/excluding T cells for assuring that double positive (CFSE+) macrophages referred to internalized and not bound apoptotic thymocytes.

☒ Tick this box to confirm that a figure exemplifying the gating strategy is provided in the Supplementary Information.
